# Nuclear Tau, p53 and Pin1 Regulate PARN-Mediated Deadenylation and Gene Expression

**DOI:** 10.3389/fnmol.2019.00242

**Published:** 2019-10-15

**Authors:** Jorge Baquero, Sophia Varriano, Martha Ordonez, Pawel Kuczaj, Michael R. Murphy, Gamage Aruggoda, Devon Lundine, Viktoriya Morozova, Ali Elhadi Makki, Alejandra del C. Alonso, Frida E. Kleiman

**Affiliations:** ^1^Chemistry Department, Hunter College and Biochemistry Program, The Graduate Center, The City University of New York, New York, NY, United States; ^2^Department of Biology and Center for Developmental Neuroscience, College of Staten Island, Graduate Center, The City University of New York, Staten Island, NY, United States

**Keywords:** mRNA processing, p53, nuclear tau, Pin1, cellular DNA damage

## Abstract

While nuclear tau plays a role in DNA damage response (DDR) and chromosome relaxation, the mechanisms behind these functions are not fully understood. Here, we show that tau forms complex(es) with factors involved in nuclear mRNA processing such as tumor suppressor p53 and poly(A)-specific ribonuclease (PARN) deadenylase. Tau induces PARN activity in different cellular models during DDR, and this activation is further increased by p53 and inhibited by tau phosphorylation at residues implicated in neurological disorders. Tau’s binding factor Pin1, a mitotic regulator overexpressed in cancer and depleted in Alzheimer’s disease (AD), also plays a role in the activation of nuclear deadenylation. Tau, Pin1 and PARN target the expression of mRNAs deregulated in AD and/or cancer. Our findings identify novel biological roles of tau and toxic effects of hyperphosphorylated-tau. We propose a model in which factors involved in cancer and AD regulate gene expression by interactions with the mRNA processing machinery, affecting the transcriptome and suggesting insights into alternative mechanisms for the initiation and/or developments of these diseases.

## Introduction

Tau is known as a microtubule-associated protein involved in a number of neurodegenerative disorders, including Alzheimer’s disease (AD; Lee et al., 2011). In neurons of AD patients, insoluble hyperphosphorylated tau protein aggregates form intracellular lesions that accumulate into neurofibrillary tangles, known as paired helical filaments (PHFs), causing neuronal death (Lu et al., 1999; Lee et al., 2011; Beharry et al., 2014). However, in normal individuals, tau is a highly soluble, non-phosphorylated protein that stabilizes microtubules in the neuronal cytoplasm (Buée et al., 2000). We have shown that hyperphosphorylated tau disrupts the microtubule system and can act as a “prion-like” protein, inducing conformational changes in normal tau, thus acting as a gain of toxic function (Alonso et al., 2016). Tau has been detected in the nuclei of neurons (Brady et al., 1995; Greenwood and Johnson, 1995; Frost et al., 2014; Bukar Maina et al., 2016), as well as of non-neuronal cells (Cross et al., 2000). While it has been shown that hyperphosphorylated tau translocates into the nucleus (Alonso et al., 2010; Sarkar et al., 2016), only dephosphorylated tau binds to and protects neuronal DNA after oxidative and mild heat stresses, indicating that tau is a key player in early stress response (Padmaraju et al., 2010; Sultan et al., 2011). Therefore, tau functions in the nucleus are regulated by phosphorylation (Qi et al., 2015). Tau can also participate in different aspects of RNA metabolism through direct association with RNA-binding proteins (RBPs; Meier et al., 2016; Vanderweyde et al., 2016; Apicco et al., 2018; Maziuk et al., 2018). Tau regulates translation through direct interaction with ribosomes (Meier et al., 2016), and contributes to the formation of stress granules (SGs; Maziuk et al., 2018). These cytoplasmic functional interactions are affected in AD (Vanderweyde et al., 2012, 2016; Meier et al., 2016; Apicco et al., 2018; Maziuk et al., 2018). Tau’s functional roles in the nucleus and the effect of tau-RBP interaction on mRNA processing have been not fully elucidated.

Nuclear RBPs can interact with target RNAs and are involved in mRNA 3′ end processing, an essential step in eukaryotic RNA metabolism that regulates the steady-state levels of mRNA transcripts under different cellular conditions by modifying their poly(A) tails (reviewed in Cevher et al., 2010; Zhang et al., 2010). These tails are crucial for proper translation and subcellular localization (Mandel et al., 2008), and their removal from mRNA transcripts is the earliest step in mRNA decay (Wilusz et al., 2001). Regulation of mRNA 3′ end processing contributes to the cells rapid response to stress during the DNA damage response (DDR). One essential mechanism to control the length of these mRNA tails is the regulation of the deadenylation reaction, the rate-limiting step in the mRNA decay pathway (Parker and Song, 2004; Mandel et al., 2008). Poly(A)-specific ribonuclease (PARN) is the main nuclear deadenylase in mammalian cells that controls poly(A) length and, consequently, gene expression (Balatsos et al., 2012; Virtanen et al., 2013). We recently demonstrated that the tumor suppressor p53 functionally interacts with PARN. While PARN keeps p53 levels low by destabilizing p53 mRNA in non-stress conditions, the UV-induced increase p53 levels results in PARN deadenylase activation, resulting in a transcription-independent regulation of gene expression (Devany et al., 2013).

Other studies have also linked p53 and some of its isoforms to tau. p53 has a neuroprotective role in a model of tau-mediated neurodegeneration, controlling the transcription of genes involved in synaptic functions (Merlo et al., 2014). Besides, while the expression of p73, a p53 isoform, lowers phosphorylated-tau levels in AD models (Wetzel et al., 2008), p44, a shorter p53 isoform, activates the transcription of different tau kinases promoting tau phosphorylation (Pehar et al., 2014). Furthermore, tau and p53 are both targets of Pin1, a peptidyl-prolyl isomerase (PPIase; Lu et al., 1999; Zacchi et al., 2002; Zheng et al., 2002; Kimura et al., 2013). Pin1 catalyzes the cis/trans isomerization of peptidyl-prolyl peptide bonds of phosphorylated serine/threonine-proline motifs, resulting in the regulation of the post-phosphorylation conformation of its substrates (Lee et al., 2011). While Pin1 is overexpressed in most human cancers (Wulf et al., 2001; Lee et al., 2011), it is depleted in the brains of AD patients, where it is sequestered into PHFs by binding to hyperphosphorylated tau (Lu et al., 1999). Pin1 accelerates the *cis* to *trans* isomerization of phosphorylated tau to prevent the accumulation of pathogenic conformations resulting in neuronal protection (Nakamura et al., 2013). Pin1 also interacts with phosphorylated p53 after DNA damage (Zacchi et al., 2002; Zheng et al., 2002), generating conformational changes in p53 that enhance its transactivation activity and the removal of HDM2 from p53 (Ryan and Vousden, 2002). Inhibition of Pin1 in mammalian cells changes p53 ubiquitination status, causing p53 proteasomal degradation (Jentsch and Siepe, 2009). Additionally, Pin1 regulates mRNA steady-state levels of different genes, such as histones, cytokines, and mRNAs within the p53 pathway (Krishnan et al., 2014).

These findings suggest a functional interplay between tau, PARN deadenylase, p53 and Pin1 following DNA damage in the nucleus. Here, we provide unexpected insights into the underlying mechanisms. Our studies show that soluble nuclear tau can associate PARN, p53 and Pin1 to regulate mRNA 3′ end processing under non-stress and DNA damage conditions. In addition, we provide evidence that both Pin1 and tau are activators of nuclear deadenylase activity in a p53-dependent manner during DDR. Lastly, tau expression induces PARN deadenylase in a p53-dependent manner, and tau phosphorylation at certain residues, as in neurological disorders such as AD (Alonso et al., 2010), abolishes this activation. Together, our results suggest that the formation of nuclear complex(es) containing tau, Pin1, p53 and PARN is (are) necessary for the control of nuclear deadenylation, as part of the rapid and efficient response to UV-induced DNA damage. Interestingly, the expression of a group of mRNAs deregulated in AD and/or cancer are targeted by Pin1, tau and PARN, further supporting overlapping functions for these factors in the nucleus. These studies are important to further elucidate the roles of these disease-related factors in the regulation of gene expression during DDR. These connections could affect cellular transcriptomes, and hence gene expression patterns; thereby suggesting insights into alternative mechanisms for the initiation and/or developments of these diseases.

## Materials and Methods

### Tissue Culture Methods and DNA Damaging Agents

HCT116, HCT116 p53-null and RKO cells were cultured and UV-treated (40 Jm^−2^) as described (Cevher et al., 2010; Nazeer et al., 2011; Devany et al., 2013). SH-SY5Y cells (American Type Culture Collection) were cultured as suggested by ATCC. HCT116 and SH-SY5Y cells were treated with 1 μM hydroxyurea (Calbiochem) for 2 h (Kleiman and Manley, 2001).

### Juglone and ATRA Treatments, CHO Cells Transfections, and Cellular Fractionation

All juglone-treated cultures were incubated with 5 μM juglone (Sigma) for 2 h as previously described (Siepe and Jentsch, 2009). All-trans retinoic acid (ATRA)-treated cultures were incubated with 1 μM ATRA (Sigma) for 72 h as previously described (Wei et al., 2015). Chinese hamster ovary (CHO) cells were cultured and transfected with PAC-GFP vectors containing as described (Alonso et al., 2010). Cellular fractionation was done in HCT116 cells (90% confluent) using a cell fractionation kit (Thermo Scientific) following manufacturer’s instructions.

### Preparation of Nuclear and Whole Cell Extracts

After different treatments, nuclear extracts (NEs) were prepared from harvested RKO, CHO, SH-SY5Y, HCT116, and HCT116 p53^−/−^ cells as described (Cevher et al., 2010; Nazeer et al., 2011; Devany et al., 2013). Whole cell extracts were prepared by harvesting cells as previously described (Fonseca et al., 2018).

### Calf Intestinal Phosphatase (CIP) Treatment

NEs were treated with calf intestinal phosphatase (CIP) as suggested by the manufacturer (New England BioLabs). Briefly, NE samples were incubated for 1 h at 37°C. CIP treatment was stopped with 2 mM EDTA and samples were further incubated for 20 min at 30°C.

### Construction of GST-Tagged Tau Variants

GST-tagged recombinant WT-Tau and PH-Tau proteins were generated by subcloning the full-length coding sequence of each variant into pGEX-2TK bacterial expression vectors using DNA recombinant techniques. These sequences were amplified by polymerase chain reaction (PCR) from pAcGFP expression plasmids previously described (Alonso et al., 2010). The primer sequences for tau variants were as follows: forward primer 5′-ATTTGGATCCATGGCTGAGCCCCG-3′; reverse primer: 5′-GGCGAATTCGTCACAAACCCTGCTT-3′. The PCRs were performed using high-fidelity F-530 Phusion DNA polymerase (FINNZYMES) following the manufacturer instructions. The PCR products were digested by BamHI and EcoRI and the resulting DNA fragments were inserted into the pGEX-2TK vector. Constructs were confirmed by sequencing (GENEWIZ).

### Purification of Recombinant Proteins

Plasmid encoding wild-type His-p53 was provided by Dr. Prives (Columbia University), plasmid encoding His-PARN was provided by Dr. Virtanen (Uppsala University), and plasmid encoding GST-Pin1 plasmid was provided by Dr. Graves (University of North Carolina at Chapel Hill). GST-tagged tau variants were prepared as explained above. All proteins were purified as previously described (Cevher et al., 2010; Devany et al., 2013). Briefly, His-fusion proteins were transformed into BL21 cells and purified by binding to and eluting from Ni-agarose columns (Millipore) as described (Nilsson and Virtanen, 2006). GST-tagged proteins were transformed into Rosetta cells and purified using glutathione-agarose columns (GE Healthcare) as described (Cevher et al., 2010; Nazeer et al., 2011; Devany et al., 2013).

### Deadenylation Assays Using NEs

^32^P-labeled L3(A_30_) substrates were prepared as described (Martinez et al., 2000). Conditions for NEs deadenylation assays using RKO, CHO, HCT116 and HCT116 p53^−/−^ cells were described (Cevher et al., 2010; Devany et al., 2013). Incubations were performed at 30°C for 2 h. Protein concentrations of the NEs were equalized by Bradford assays (Bio-Rad) before used in deadenylation reactions.

### *In vitro* Deadenylation Assays

Conditions for *in vitro* deadenylation assays were previously described (Cevher et al., 2010). Deadenylation reactions with His-PARN, His-p53, GST-WT-Tau, or GST-PH-Tau were incubated at 30°C for 30 min.

### IP and Western Blot Analysis

Total protein (100 μg) from indicated NEs was e-IPed with antibodies against Pin1 polyclonal (H-123, Santa Cruz Biotechnology), tau polyclonal (H-150, Santa Cruz Biotechnology), PARN polyclonal (H-105, Santa Cruz Biotechnology), p53 monoclonal (SC-126, Santa Cruz Biotechnology), and p53 polyclonal (FL-393, Santa Cruz Biotechnology) as described (Cevher et al., 2010; Nazeer et al., 2011). For tau detection in Western blot analysis, tau-13 and tau-1, which is reactive when Ser198/199/202 are not phosphorylated, mouse monoclonal antibodies were used (Alonso et al., 2010). Other antibodies in Western blot analysis: Topo II (H-8, Santa Cruz Biotechnology), PARN (A303-562A, Bethyl), Actin (A2066, Sigma), Pin1 (A302-316A, Bethyl), α-tubulin (2871-1, Epitomics), EGFR (1005, Santa Cruz Biotechnology), and H_2_B (8135, Cell Signaling). Because antibody tau-1 recognizes tau only when it is not phosphorylated, the blots were pretreated with alkaline phosphatase (196 units/ml) in 0.1 M Tris, pH 8.0, and 1 mM phenylmethylsulfonyl fluoride for 5 h prior to incubation with the primary antibody (Alonso et al., 2001).

### Protein-Protein Interaction Assays

The pull-down assays with GST-Pin1, GST-tagged tau derivatives, His-p53 and His-PARN were performed as described (Cevher et al., 2010; Nazeer et al., 2011; Devany et al., 2013). two-hundred microgram weight of total protein from each NE was pulled-down with 2 μg of the indicated GST- or His-fusion proteins-bound beads, the binding and washing conditions were as described (Cevher et al., 2010; Nazeer et al., 2011; Devany et al., 2013). NEs were treated with 50 mg of RNase A per ml for 10 min at 4°C.

### siRNA-Mediated Knockdown of Pin1, Tau, PARN, and p53

On-Targetplus Smartpool siRNAs specific for human Pin1, PARN, p53, and control siRNA duplexes were obtained from Dharmacon RNA technologies and used as before (Cevher et al., 2010; Nazeer et al., 2011; Devany et al., 2013; Zhang et al., 2015). siRNA specific for human tau was obtained from Ambion-Life Technologies. siRNA treatments were as described (Devany et al., 2013). Knockdown of proteins was confirmed by Western blot analysis using the antibodies indicated in each figure.

### Overexpression of Tau Derivatives

pAcGFP expression plasmids containing WT-Tau or PH-Tau sequences and described before (Alonso et al., 2010) were transfected into HCT116 cells as previously described (Devany et al., 2013). Overexpression was confirmed by Western blot analysis using the antibodies indicated in each figure.

### Real-Time Reverse Transcription Polymerase Chain Reaction (qRT-PCR) of Endogenous mRNAS

As described before (Cevher et al., 2010; Devany et al., 2013), equivalent amounts (2 μg) of purified nuclear RNA from HCT116 cells were used as a template to synthesize cDNA with oligo-d(T) primers using qScript cDNA SuperMix (Quanta Bioscience) according to the manufacturer’s protocol. Commercially available primers for *ANXA1* (NM_000700), *FOS* (NM_005252), *BIRC3* (NM_001165), *NOTCH1* (NM_017617) and pleomorphic adenoma gene-like 2 (*PLAGL2*; NM_002657) were used in the qRT–PCR reactions (Integrated DNA Technologies). *UBC*: forward primer 5′-TGGCACAGCTAGTTCCGTCGCA-3′, reverse 5′-CGAGGGTGATGG TCTTACCAGTC-3′. *MAPT*: forward primer 5′-GGCAACATCCATCATAAACCAGGA-3′, reverse 5′-GGTCAGCTTGTGGGTTTCAATC TT-3′. *TP53*: forward primer 5′-GCCTGAGGTTGGCTCTGACTGTA-3′, reverse 5′-CCTCAAAGCTGTTCCGTCCC AGTA-3′. Relative levels were calculated using the ΔΔC_T_ method as before (Cevher et al., 2010; Devany et al., 2013), with RT products of *UBC* from RNA samples from control conditions used as endogenous control.

### Animals

Mice expressing in the neurons PH-Tau (Di et al., 2016), WT-Tau (Andorfer et al., 2003) and non-transgenic mice from 15 to 24 months were anesthetized and transcardially perfused sequentially with 0.1 M phosphate buffered saline (PBS) and 4% paraformaldehyde in 0.1 M PBS or 1% paraformaldehyde and 1% glutaraldehyde in 0.1 M PBS (pH 7.4; Sarsoza et al., 2009). Brains were removed and further fixed by immersion with the same solution above at 4°C for 1 week. Cryosections in the coronal plane (40 μm) were cut on a cryostat and stored at −20°C in a solution with 30% Ethylene Glycol and Sucrose in 0.1 M PBS. Coronal vibratome sections (50 μm) were cut and stored at 4°C in 0.1 M PBS.

### Immunofluorescence

A PBS-0.2% Triton X-100 (PBST, Sigma Chemical. Co., St. Louis, MO, USA) solution was used in all washing steps. Free floating sections were placed in wells of 24-well plates and were rinsed for 10 min in PBST and blocked for 60 min with blocking buffer (BB) containing 10% Normal Goat Serum-10% Normal Horse Serum in PBST. Slices were then incubated overnight at 4°C under slight agitation with primary antibody dissolved in BB. Next day, slices were incubated for 2 h at room temperature in the dark with AlexaFluor 488 conjugated donkey anti-rabbit or mouse IgG (1:500, Invitrogen Life Sciences, Carlsbad, CA, USA) secondary antibody diluted in BB. After washings, slices were incubated with 0.001% Topro III dissolved in PBST for 25 min, after extensive washings slices were mounted onto gelatin-coated slides using Vectashield hard set with DAPI (Vector Laboratories) as a mounting medium. Slices were observed under a LEICA TGS SP5 confocal laser-scanning microscope (Leica Microsystems CMS GmbH, Mannheim, Germany). Confocal scans were taken at 2 μm z-steps keeping all parameters (pinhole, contrast and brightness) constant. Image analyses were conducted on image z-stacks which contained the field of interest. Images were assembled into montages using Adobe Photoshop (Adobe Systems, Mountain View, CA, USA). Immunohistochemical staining controls were performed by omitting the primary or secondary antibodies to confirm the specificity of the staining.

### RACE-Poly(A) Test Assays

Nuclear RNA from HCT116 cells treated with either PARN, Tau or control siRNA was reverse-transcribed using oligo (dT)-anchor primer (5′-GGGGATCCGCGGTTTTTTTTTT-3′) and GoScript Reverse Transcriptase (Promega). One microliter of each cDNA was used for PCR amplification by GoTaq PCR mix (Promega) using *ANXA1* 3′UTR-specific primer located 95 bp upstream of the poly(A) site and oligo (dT)-anchor primer.

## Results

### Tau Interacts With Pin1 and Factors Involved in mRNA 3′ Processing in the Nucleus to Form (a) Complex(es)

**Figure 1:**
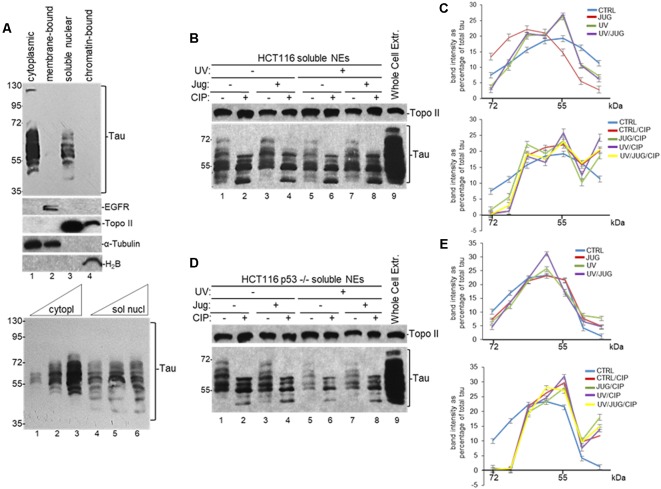
Distribution of nuclear tau forms changes under different cellular conditions. **(A)** Different tau forms are present in cytoplasmic and soluble nuclear fractions from HCT116 cells. Equivalent amounts of total protein content from each sample obtained by subcellular fractionation kit were analyzed by SDS-PAGE. Tau forms were detected using monoclonal tau-13 antibody (Alonso et al., 2010). Antibodies against epidermal growth factor receptor (EGFR), topoisomerase II (Topo II), α-Tubulin, and histone H_2_B were used as controls for each fraction. Bottom panel: increasing amounts of cytoplasmic (5, 15 and 30 μg of total protein) and soluble nuclear (30, 45 and 60 μg of total protein) fractions were loaded to detect different tau forms. A representative Western blot from three independent biological assays is shown. **(B–E)** Distribution of nuclear tau forms changes upon Pin1 inactivation, p53 expression, UV irradiation and calf intestinal phosphatase (CIP) treatment. **(B)** HCT116 and **(D)** HCT116 p53^−/−^ cells were treated with juglone (5 μM) for 2 h, and/or exposed to UV irradiation (40 Jm^−2^) and allowed to recover for 2 h before nuclear extracts (NEs) were prepared. NEs were treated with CIP as indicated. Samples were analyzed by immunoblotting with the indicated antibodies. Antibody against Topo II was used as loading control. Whole-cell extracts were also analyzed. A representative Western blot from three independent biological assays is shown. **(C,E)** Band intensities of tau forms detected between 72- and 35-kDa in **(B,D)**, respectively, were plotted as percentage of total tau expression in each condition. Blots were arbitrarily divided into seven regions for quantification. The data shown are mean ± SEM from three independent biological experiments. Quantifications were done using ImageJ software (http://rsb.info.nih.gov/ij/).

Various studies have shown that tau localizes to the nuclei of neurons (Brady et al., 1995; Greenwood and Johnson, 1995; Frost et al., 2014; Bukar Maina et al., 2016), and non-neuronal cells (Cross et al., 2000); and it can interact with different RBPs participating in diverse RNA metabolic pathways (Meier et al., 2016; Vanderweyde et al., 2016; Apicco et al., 2018; Maziuk et al., 2018). Nuclear RBPs can regulate mRNA 3′ end processing, an essential step in eukaryotic RNA metabolism, by selectively interacting with target RNAs resulting in the regulation of the steady-state levels of those transcripts under different cellular conditions (Matoulkova et al., 2012). Together, those studies suggest that tau might play a role in the nucleus by interacting with different RBPs to regulate mRNA processing. To analyze this possibility, we first examined the presence of tau in the nucleus of human colorectal carcinoma HCT116 cells using different cellular fractions. Consistent with previous studies (Cross et al., 2000), we detected tau in both cytoplasmic and soluble nuclear fractions (Figure 1A). While both cytoplasmic and nuclear fractions show similar forms of tau, our Western blot analysis revealed that some of these forms were exclusively present in one compartment or the other.

To further analyze the nature and possible posttranslational modifications of these tau forms, we analyzed NEs from HCT116 cells exposed to various treatments. Because nuclear tau is involved in DDR (Padmaraju et al., 2010; Sultan et al., 2011), we tested the effect of UV treatment on tau forms pattern. Additionally, as tau functionally overlaps with p53 (Wetzel et al., 2008; Pehar et al., 2014; Merlo et al., 2014), we also analyzed these tau forms with tau-13 antibody in samples from isogenic HCT116 p53^−/−^ cells. Furthermore, since tau is a substrate of PPIase Pin1 (Lu et al., 1999; Kimura et al., 2013), we treated cells with 5-hydroxy-1,4-naphthoquinone (juglone), an inhibitor of Pin1 activity (Chao et al., 2001) that has been used to study the role of Pin1 in different cellular processes (Siepe and Jentsch, 2009). Western blot analysis revealed that the total amount of tau (Supplementary Figure S1A) and the distribution of nuclear tau forms changed in each condition depending on DNA damage induction, p53 expression, and/or Pin1 activity (Figures 1B–E). After treatment with CIP, some of the high molecular weight forms of tau change their migration rate, indicating that some of the nuclear tau forms are phosphorylated. Thus, our data suggest that these dynamic changes in the distribution of tau forms, some of which are generated by posttranslational modifications, might be associated to some nuclear functions during DDR.

**Figure 2:**
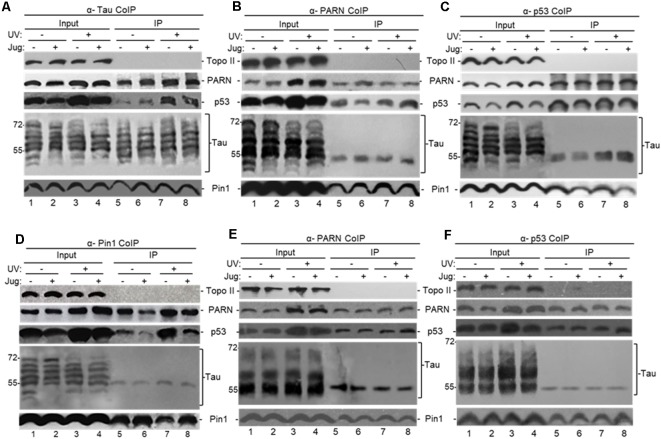
Tau interacts with factors involved mRNA 3′ end processing to form (a) complex(es). **(A–F)** Tau can form (a) complex(es) with Pin1, p53 and poly(A)-specific ribonuclease (PARN) in NEs of HCT116 cells independently of DNA damaging conditions. Endogenous-immunoprecipitation (e-IP) assays with antibodies against tau **(A)**, PARN **(B,E)**, p53 **(C,F)** or Pin1 **(D)** were performed using NEs of HCT116 cells treated with juglone (5 μM) for 2 h, and/or exposed to UV irradiation (40 Jm^−2^) and allowed to recover for 2 h. NEs were treated with RNase A. Equivalent amounts of the pellets (IP) were resolved by SDS-PAGE, and proteins were detected by Western blot using the indicated antibodies. **(A–D)** Tau-13 mouse monoclonal antibody was used for Western blot detection. **(E,F)** Nitrocellulose membranes were dephosphorylated before tau-1 use in Western blot detection. Ten percentage of the NEs used in the e-IP assays are shown as input. Representative e-IP reactions from three independent biological assays are shown.

To identify proteins that can interact with nuclear tau we performed endogenous-immunoprecipitation (e-IP) assays using anti-tau polyclonal antibodies and NEs from HCT116 and the isogenic HCT116 p53^−/−^ cells incubated with juglone and/or UV treated. When analyzed with tau-13 antibody, the inputs in Figure 2, Supplementary Figure S1B and Figure 1B revealed similar changes in the distribution of nuclear tau forms in the conditions studied. Consistent with previous studies (Lu et al., 1999; Kimura et al., 2013), our e-IPs indicated that nuclear tau can form (a) complex(es) with p53 and the PPIase Pin1 and that these interactions were not affected by DNA damaging conditions or Pin1 activity (Figure 2A). Interestingly, as part of the tau-containing complexes, we detected nuclear PARN deadenylase, which we previously showed can form (a) complex(es) with p53 resulting in activation of PARN deadenylase (Devany et al., 2013). In the reciprocal e-IP experiments using antibodies against either PARN (Figure 2B), p53 (Figure 2C) or Pin1 (Figure 2D), we detected forms of nuclear tau with molecular weight around 55 kDa IPed by each antibody. When tau-1 antibody, which is reactive with tau non-phosphorylated at Ser198/199/202, was used for the blot analysis nuclear tau forms of molecular weight around 55 kDa were also detected in the e-IP (Figures 2E,F). Besides, consistent with Figure 2B, Pin1 interaction with tau nuclear form of molecular weight around 55 kDa was not detected in samples from tau depleted cells (Supplementary Figure S1C). Interestingly, a pathological tau form of 55 kDa generated from alternative splicing has been described (Sergeant et al., 1997). Besides 55 kDa tau forms have been recognized in Pick’s disease (Sergeant et al., 1997) and in cerebrospinal fluid from patients with neurodegenerative disorders (Borroni et al., 2009). Interestingly, a 55 kDa tau form with impediments to dimerize was identified in rat glaucoma models (Chiasseu et al., 2016). While more studies are needed to characterize the tau form at 55 kDa that binds to Pin1 and other members of the PARN complex, it is possible that this a tau form less prone to form dimers and is more available for binding to other nuclear factors in HCT116 cells.

We also treated HCT116 cells with ATRA, an inhibitor of Pin1 activity and expression (Wei et al., 2015) that has been used as potential cancer therapeutic agent (Nakatsu et al., 2018; Yang et al., 2018; Zhang et al., 2019). As with juglone, NEs from HCT116 cells incubated with ATRA showed the formation of (a) complex(es) containing p53, PARN and a nuclear tau form of around 55 kDa (Supplementary Figure S1D). As all samples were treated with RNase A, these observed interactions were probably not due to an RNA tethering effect. Our studies also indicate that tau, PARN and Pin1 can co-immunoprecipitate independently of p53 expression in NEs from HCT116 under non-stress conditions (Figure 2) and in NEs from HCT116 p53^−/−^ cells (Supplementary Figure S1B). As expected, the amount of p53 e-IPed by nuclear tau increased after the UV-induction of p53 expression (Nazeer et al., 2011; Devany et al., 2013). These results indicate that soluble nuclear tau can associate with PARN, p53 and Pin1 under non-stress and DNA damage conditions, and the nature and function of those complex(es) might be different under each cellular condition and dependent on the presence of p53.

**Figure 3:**
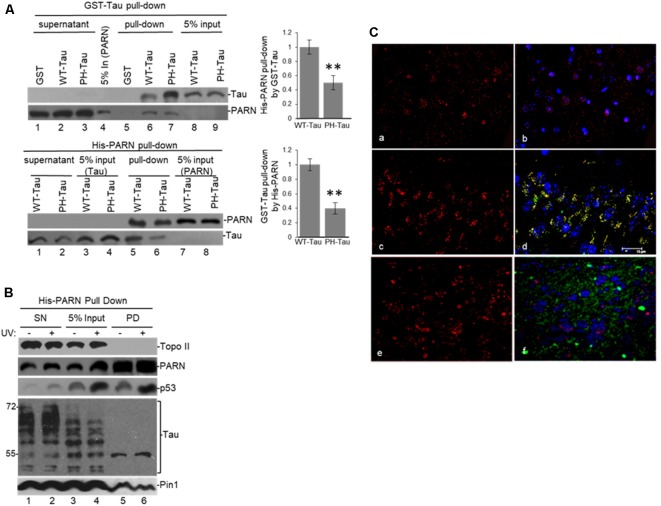
**(A)** WT-Tau interacts with PARN, and the interaction is reduced by tau phosphorylation at pathological sites. Immobilized GST-WT-Tau or GST-PH-Tau, a tau variant with phosphorylation at pathological sites, on glutathione beads were incubated with His-PARN (top panel). Alternatively, immobilized His-PARN on nickel beads was incubated with GST-WT-Tau or GST-PH-Tau (bottom panel). Tau variants and His-PARN constructs were previously described (Alonso et al., 2010; Cevher et al., 2010). A representative pull-down reaction from three independent assays is shown. Equivalent amounts of the pulldowns (PD) and supernatants (SN) were analyzed and proteins were detected by immunoblotting with the indicated antibodies. Five percent of the proteins used in the PD assays are shown as input. On the left, relative binding (RB) of PH-Tau/His-PARN and WT-Tau/His-PARN was plotted. Binding of WT-Tau to His-PARN was arbitrarily set at 1.0. The data used are the mean RB ± SEM from three independent biological assays. Quantifications were done using ImageJ software (http://rsb.info.nih.gov/ij/). The *P*-values are indicated as **(≤0.005). **(B)** Immobilized His-PARN on nickel beads was incubated with NEs from UV-treated or untreated HCT116 cells. A representative pull-down reaction from three independent assays is shown. Equivalent amounts of the pulldowns (PD) and supernatants (SN) were analyzed and proteins were detected by immunoblotting with the indicated antibodies. Five percent of the NE used in the pull-down reactions is shown as input. **(C)** Coronal slices of hippocampus (CA3) stained with polyclonal PARN antibodies (red) showed changes in PARN distribution in mice expressing human PH-Tau (green). Non-transgenic mice **(a,b)** and WT-Tau expressing mice **(e,f)** showed PARN staining mainly associated to the nucleus, whereas the mice expressing human PH-Tau **(c,d)** showed accumulation of PARN in the perinuclear area co-localizing with PH-Tau. **(a,c,e)**: only PARN staining; **(b,d,f)**: merged images (PARN, PH-Tau and DAPI for the nuclei). Monoclonal human tau antibodies were used.

To further examine the interaction between tau and PARN deadenylase we performed “pull-down” assays using full-length PARN (His-PARN) and full-length WT-tau (GST-WT-tau; Figure 3). We also analyzed PARN interaction with a pseudo-phosphorylated-tau mutant derivative (PH-Tau). PH-Tau is a phosphomimetic mutant with Ser199, Thr212, Thr231 and Ser262 residues changed to glutamic acid, and hence resembles a pathological human form of the protein found in neurological diseases, such as AD (Beharry et al., 2013). Our data showed that PARN interacted directly with WT-Tau, and this interaction was weaker with PH-Tau (Figure 3A). Consistent with the e-IPs (Figure 2 and Supplementary Figure S1B), His-PARN pulled-down Pin1 and p53 from NEs from HCT116 cells in non-stress conditions and after UV treatment (Figure 3B), as well as nuclear tau forms of molecular weight around 55 kDa. Similar results were obtained with His-p53 (Supplementary Figure S2A), GST-Tau (Supplementary Figure S2B) or GST-Pin1 (Supplementary Figure S2C) pull-downs. Interestingly, when PH-Tau was used instead of WT-Tau, the complex formation with endogenous nuclear PARN present in the extracts was not detected (Supplementary Figure S2B), suggesting that changes in either tau phosphorylation at pathological sites or in PARN might affect their interaction. To test whether PH-Tau and PARN co-localize *in vivo*, we analyzed coronal brain slices of mice expressing WT-Tau (Andorfer et al., 2003), PH-Tau (Di et al., 2016), and non-transgenic mice. The brain slices were stained with a polyclonal antibody against PARN and a monoclonal antibody against human tau. In hippocampus (CA3) of non-transgenic and WT transgenic mice, PARN staining was detected mainly associated to the nucleus (Figures 3C,a,b,e,f). PARN staining increased in the cytoplasm of PH-Tau transgenic mice (Figures 3c,d), with a marked accumulation of PARN in the perinuclear area and co-localization with PH-Tau staining (Figures 3C,c,d). This is consistent with Vanderweyde et al.’s (2016) observations that the overexpression of abnormal tau results in its accumulation in cytosolic SGs where it interacts with RBPs. Our results suggest that PH-Tau might capture PARN deadenylase in these cytosolic aggregates composed of proteins and RNAs. Together, these results provide evidence that tau can associate with factors previously described to be involved in mRNA 3′ processing, such as tumor suppressor p53, Pin1 and PARN deadenylase, in the nucleus, and that phosphorylation of tau at pathological sites might affect the formation of those complexes.

### Tau Expression Levels and Phosphorylation Can Regulate PARN Deadenylase Activity in a p53-Dependent Manner

**Figure 4:**
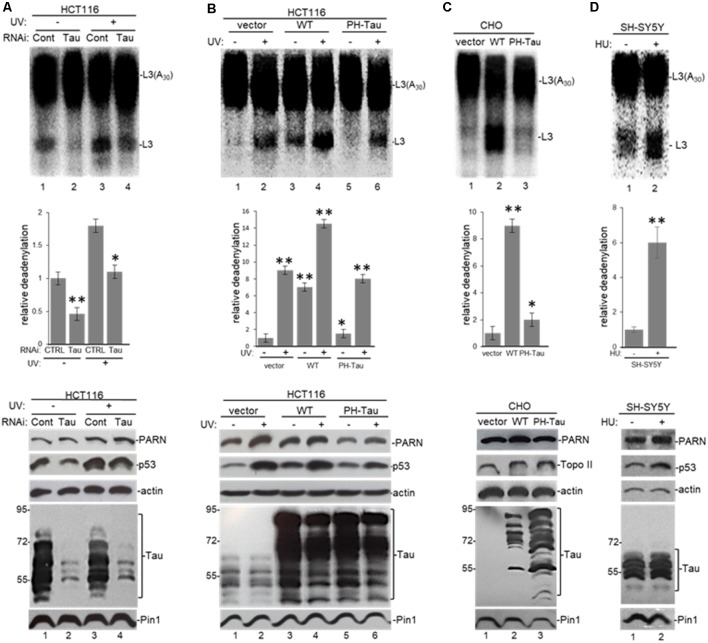
Tau expression and phosphorylation regulates nuclear deadenylase activity. **(A)** Tau depletion inhibits nuclear deadenylation under non-stress and UV-induced conditions in HCT116 cells. NEs were prepared from HCT116 cells treated with either *MAPT* or control siRNAs for 48 h, and/or treated with UV (40 Jm^−2^) and allowed to recover for 2 h. NEs and radiolabeled capped L3(A_30_) RNA substrate were used in deadenylation assays. Deadenylation reactions were incubated for 90 min (Cevher et al., 2010). RNAs were purified and analyzed by denaturing PAGE. Representative deadenylation reactions from three independent biological assays are shown. Positions of the polyadenylated RNA L3(A_30_) and the L3 deadenylated product are indicated. On the right, relative deadenylation (RD) levels, calculated as {L3 fragment/[L3 fragment + L3(A_30_)]} × 100, were plotted for each condition. The RD level for samples from control siRNA treated cells was arbitrarily set at 1.0. The means ± standard deviation of RD values are indicated. Quantifications were done using ImageJ software (http://rsb.info.nih.gov/ij/). The *P*-values are indicated as *(≤0.05) or **(≤0.005). Lower panels show expression levels of indicated proteins from NEs tested in deadenylation assays. **(B,C)** Expression of WT-Tau but not of its phospho-derivative PH-Tau induces nuclear deadenylase activity in **(B)** HCT116 and **(C)** chinese hamster ovary (CHO) cells. NEs from cells transfected with pAc-GFP expression vectors containing either WT-Tau or PH-Tau (described in Figure 3; Alonso et al., 2010) were used in deadenylation assays and analyzed as described in **(A)**. Additionally, HCT116 cells were treated with UV (40 Jm^−2^) and allowed to recover for 2 h. The RD level for samples treated with the only vector was arbitrarily set at 1.0. **(D)** Nuclear deadenylation is induced in SH-SY5Y cells treated with hydroxyurea (HU). NEs from untreated SH-SY5Y cells or cells treated with 1 μM HU for 2 h were used in deadenylation assays and analyzed as described in **(A)**. The RD level for untreated samples was arbitrarily set at 1.0.

To study the significance of the interaction between nuclear tau and factors involved in mRNA 3′ end processing, we performed deadenylation assays using samples from HCT116 cells depleted in tau expression by siRNA-mediated treatment under different cellular conditions. NEs from those cells were assayed for deadenylation activity using a radiolabeled L3(A_30_) RNA substrate as described (Cevher et al., 2010; Devany et al., 2013). Consistent with our previous studies (Devany et al., 2013), Figure 4A shows that deadenylation activity in NEs of HCT116 cells treated with control siRNA increased after UV treatment (lanes 1 and 3). *MAPT* siRNA treatment resulted in substantial depletion of tau nuclear forms in HCT116 cells without an obvious effect on PARN levels. Interestingly, siRNA-mediated knockdown of tau resulted in a decrease in p53 levels both before and after UV. Samples from cells exposed to UV and either control or *MAPT* siRNA showed the previously described increase in deadenylation (Cevher et al., 2010), probably due to the UV-induced increase in p53 levels, which is another activator of PARN-mediated deadenylation (Devany et al., 2013). Importantly, tau depletion inhibited deadenylation before and after UV exposure (Figure 4A), suggesting that tau levels might play a role in the activation of deadenylation in human cells. Alternatively, siRNA-treated cells were exposed to hydroxyurea (HU), a reversible ribonucleotide reductase inhibitor that mimics DNA damage. Consistent with our previous studies (Kleiman and Manley, 2001), HU-treatment had similar effects on deadenylation to those observed with UV irradiation (Supplementary Figure S3A). Supporting these results, the analysis of NEs from HCT116 cells transfected with a pAcGFP expression construct for WT-Tau showed activated nuclear deadenylation in both non-stress conditions (Figure 4B, lanes 1 and 3) and under UV treatment (lanes 2 and 4). Importantly, this strong activation was not observed in NEs of cells expressing empty vector or PH-Tau, the phosphomimetic mutant that resembles a pathological human form of the protein found in neurological diseases. This lack of activation of nuclear deadenylation by PH-Tau overexpression might reflect the weaker interaction of PH-Tau with PARN observed in Figure 3A and Supplementary Figure S2B.

Extending these studies, the overexpression of WT-Tau, but not of PH-Tau, in CHO cells, which normally do not express tau (Matsumura et al., 1999) or p53 (Moro et al., 1995), induced nuclear deadenylase activity (Figure 4C). Using SH-SY5Y neuroblastoma cells exposed to HU to mimic DNA damaging conditions (Kleiman and Manley, 2001), we also observed the induction of nuclear deadenylation activity (Figure 4D). Interestingly, HU can be transported across the blood-brain barrier (Dogruel et al., 2003) and can regulate oxidative and metabolic stress and improve memory in the mouse model of AD (Brose et al., 2018). Besides, and as shown in Figure 3C, it is possible that PH-Tau overexpression might capture PARN in cytosolic SGs, decreasing the levels of nuclear PARN (Figure 4B, compare lanes 3–4 to 5–6) and the deadenylation levels. In fact, cytosolic PARN levels (62 kDa isoform; Cevher et al., 2010) were enriched in cytoplasmic fractions from cells expressing PH-Tau but not in samples from cells expressing WT-Tau (Supplementary Figure S3B). Interestingly, a decrease in nuclear PARN (74 kDa isoform) was observed in samples from cells expressing PH-Tau. Importantly, the WT-Tau-mediated activation of deadenylation observed in nuclear samples from HCT116 cells was lower when samples from isogenic p53-null HCT116 cells were analyzed (Supplementary Figure S3C). These results indicate that p53 expression is necessary for the full tau-mediated activation of nuclear deadenylation and supports the idea of functional overlapping of both proteins on the regulation of mRNA 3′ processing. Together, these results indicate that tau functionally overlaps p53 in the activation of deadenylation in the nucleus and that tau phosphorylation at Ser199, Thr212, Thr231 and Ser262 residues regulates those functions.

**Figure 5:**
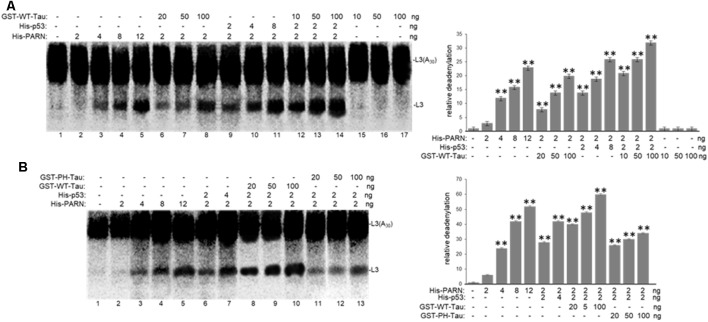
WT-Tau but not PH-Tau phospho-variant can activate PARN-dependent deadenylation *in vitro*, and this activation is stronger in the presence of p53. *In vitro* deadenylation assays (Cevher et al., 2010) were performed using different concentrations of His-PARN and His-p53, and increasing amounts of **(A)** GST-WT-Tau or **(B)** either GST-WT-Tau or GST-PH-Tau. Deadenylation assays were performed in the presence of radiolabeled capped L3(A_30_) RNA substrates. RNAs from reactions were analyzed as in Figure 4. Deadenylation reactions were incubated for 90 min. RNAs were purified and analyzed by denaturing PAGE. Representative reactions from three independent biological assays are shown. Positions of the polyadenylated RNA L3(A_30_) and the L3 deadenylated product are indicated. On the right, relative deadenylation (RD) levels, calculated as {L3 fragment/[L3 fragment + L3(A_30_)]} ×100, were plotted for each condition. The levels for control conditions with no recombinant proteins added were arbitrarily set at 1.0. The means ± standard deviation of RD values are indicated. The *P*-values are indicated as **(≤0.005). Quantifications were done using ImageJ software (http://rsb.info.nih.gov/ij/).

To further investigate the role of tau in deadenylation we tested whether tau was able to regulate PARN activity directly. We performed *in vitro* reconstituted deadenylation assays using limiting amounts of His-PARN in a cell-free assay and increasing amounts of different GST-tagged recombinant tau variants. Consistent with results shown in Figure 4, GST-WT-Tau induced His-PARN deadenylation activity in a concentration-dependent manner (Figure 5A, lanes 6–8). As previously described (27), His-p53 was also able to activate PARN (lanes 9–11). Interestingly, the activation of PARN-mediated deadenylation by GST-WT-Tau was further increased when a limiting amount of His-p53 was also included in the reaction (lanes 12–14), indicating that these two factors have an additive effect on the regulation of mRNA 3′ processing. GST-PH-Tau was not able to activate PARN deadenylation as strongly as WT-Tau (Figure 5B, compare lanes 11–13 to 8–10), indicating that tau phosphorylation plays an important role in the tau-mediated regulation of PARN activity. GST-WT-Tau derivative was not able to deadenylate the substrate in the absence of His-PARN (Figure 5A, lanes 15–17). Together these results indicate that tau and p53 have an additive effect on the activation of deadenylation by PARN. Although our data does not uncover the mechanism(s) involved in this cellular response, it is possible that the tau-induced activation of PARN deadenylase activity could be the result of several alternative mechanisms such as a direct interaction between tau, PARN and p53 and/or by the formation of any other activator complex.

### The Prolyl Isomerase Pin1 Is Part of a Deadenylation Activator Complex That Includes PARN, Tau and p53 Under Damaging Conditions

**Figure 6:**
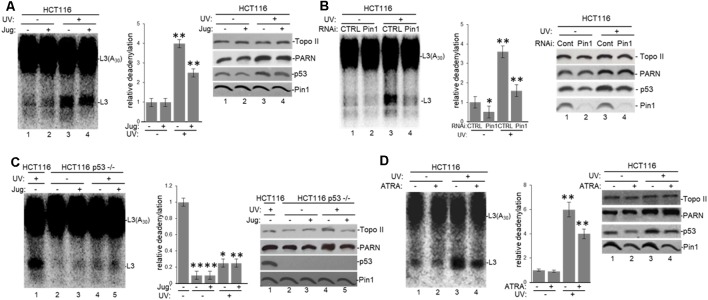
Pin1 is part of a deadenylation activator complex that includes PARN, tau and p53 under damaging conditions.** (A,B)** Levels of nuclear deadenylation correlate with Pin1 activity and expression in HCT116 cells. NEs from cells incubated with either **(A)** 5 μM juglone for 2 h or **(B)** Pin1/control siRNAs for 48 h were used in deadenylation assays. Indicated samples were also treated with UV (40 Jm^−2^) and allowed to recover for 2 h. The deadenylation reactions were incubated for 90 min. RNAs were purified and analyzed by denaturing PAGE. Representative deadenylation reactions from three independent biological assays are shown. Positions of the polyadenylated RNA L3(A_30_) and the L3 deadenylated product are indicated. On the right, relative deadenylation (RD) levels, calculated as {L3 fragment/[L3 fragment + L3(A_30_)]} ×100, were plotted for each condition. The RD levels for control conditions were arbitrarily set at 1.0. The means ± standard deviation of RD values are indicated. The *P*-values are indicated as *(≤0.05) or **(≤0.005). Quantifications were done with ImageJ software (http://rsb.info.nih.gov/ij/). Right panels show Western blot analysis of indicated proteins from NEs tested for deadenylation. **(C)** Pin1-mediated activation of nuclear deadenylation is dependent on p53 expression. NEs from HCT116 or HCT116 p53^−/−^ cells incubated with 5 μM juglone and/or treated with UV (40 Jm^−2^) and allowed to recover for 2 h were used in deadenylation assays. Deadenylation reactions were performed as described in **(A,B)**. RD levels in samples from HCT116 cells were arbitrarily set at 1.0. Right panels show Western blot analysis of indicated proteins from NEs used in deadenylation assays. **(D)** Levels of nuclear deadenylation were affected by Pin1-inhibitor all-trans retinoic acid (ATRA) in HCT116 cells. NEs from cells incubated with 1 μM ATRA for 72 h were used in deadenylation assays. Indicated samples were also treated with UV (40 Jm^−2^) and allowed to recover for 2 h. Deadenylation reactions were performed as described in **(A)**. RD level for untreated HCT116 cells was arbitrarily set at 1.0. Right panels show Western blot analysis of indicated proteins from NEs used in deadenylation assays.

To further analyze the tau-mediated activation of deadenylation in the nucleus, we tested the role of Pin1 which is involved in both tau and p53 isomerization of peptidyl-prolyl peptide bonds when phosphorylated (Lu et al., 1999; Zacchi et al., 2002; Zheng et al., 2002; Kimura et al., 2013), in this reaction. Deadenylation assays using NEs from HCT116 (Figure 6A) and colon carcinoma RKO cells (Supplementary Figure S4A) treated with juglone revealed that Pin1 inactivation inhibited nuclear deadenylation independently of stress conditions. These results suggest that Pin1, like tau and p53, plays a role in the activation of nuclear deadenylation. While juglone treatment did not cause a considerable change in Pin1 and PARN expression levels, it caused, as previously described (Zacchi et al., 2002; Zheng et al., 2002), a decrease in p53 levels. In addition, siRNA-mediated knockdown of Pin1 in HCT116 cells decreased nuclear deadenylation activity in both non-treated and in UV-treated HCT116 cells (Figure 6B), suggesting that Pin1 levels might activate deadenylation. Interestingly, deadenylation was not affected by the inactivation of Pin1 in the isogenic HCT116 p53^−/−^ cell line (Figure 6C); indicating that Pin1 effect on nuclear deadenylation depends on p53 expression. While Pin1, PARN and tau can form (a) complex(es) in samples independently of p53 expression (Figure 2 and Supplementary Figure S1B), p53 expression is necessary for the optimal activation of nuclear deadenylation by Pin1/PARN/tau (Figure 6C). Inhibition of nuclear deadenylation, especially during stress conditions, was also observed when ATRA, an inhibitor of Pin1 (Wei et al., 2015), was used instead of juglone (Figure 6D), indicating that this prolyl isomerase has a specific role of in the regulation of 3′ processing. Addition of either juglone or ATRA to the deadenylation reaction (Supplementary Figure S4B) did not have non-specific inhibitory effects, as they only inhibited deadenylation when added to cell cultures where they have a specific regulatory effect on Pin1. Our data suggest that Pin1 might regulate deadenylation by catalyzing the cis/trans isomerization of peptidyl-prolyl peptide bonds of phosphorylated p53 and/or tau serine/threonine-proline motifs (Lu et al., 1999; Zacchi et al., 2002; Zheng et al., 2002; Kimura et al., 2013), resulting in the regulation of the post-phosphorylation conformation and, hence, function (Lee et al., 2011). This regulatory mechanism might be relevant in conditions where the amount of p53 increases, such as under stress conditions.

### The Deadenylation Activator Complex Pin1/Tau/p53/PARN Is Involved in the Degradation of a Group of Transcripts Deregulated in AD and/or Cancer

The data presented above provide evidence that tau is part of a deadenylation activator complex in the nucleus, suggesting that PARN, Pin1 and tau might have some common mRNA targets that are regulated by these factors under different cellular conditions. We previously showed that PARN is involved in the degradation of different transcripts under different cellular conditions (Cevher et al., 2010; Devany et al., 2013). Our studies indicated that PARN plays a role in decreasing the levels of short-lived mRNAs involved in the control of cell growth and differentiation, keeping their expression levels low under non-stress conditions. Furthermore, it was described that Pin1 regulates mRNA levels of genes with short half-lives by using microarray assays on nuclear RNA samples from HeLa cells (Krishnan et al., 2014). To find common mRNA targets of Pin1 and PARN deadenylase we compared two studies that used depletion of those factors followed by microarray analysis (Devany et al., 2013; Krishnan et al., 2014). As PARN is a deadenylase, we focused our search on target mRNAs that showed an increase in the steady-state levels by inhibition of the potential PARN-mediated pathway. When we compared the target genes in both studies, we identified 8 transcripts that were upregulated by the knockdown of both PARN (Devany et al., 2013) and Pin1 (Krishnan et al., 2014) under non-damaging conditions (Supplementary Figure S5A).

**Figure 7:**
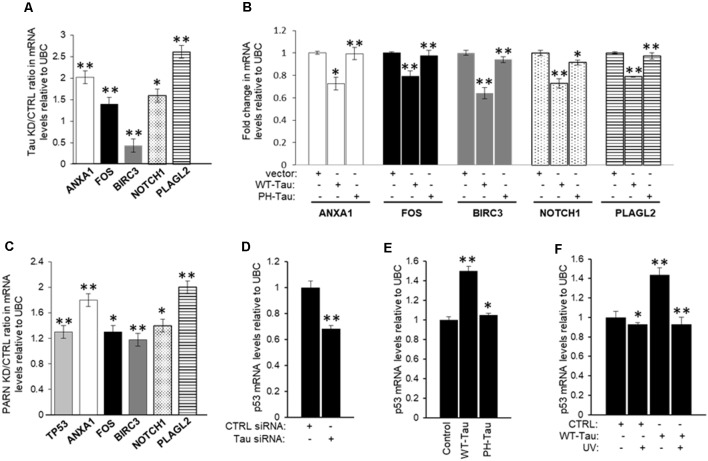
Expression of a group of mRNAs deregulated in cancer and/or Alzheimer’s disease (AD) is targeted by tau, Pin1 and PARN. **(A)** Effect of tau knockdown on endogenous gene expression of selected targets. Real-time reverse transcription polymerase chain reaction (qRT-PCR) analysis of *ANXA1*, *FOS*, *BIRC3*, *NOTCH1*, and pleomorphic adenoma gene-like 2 *(PLAGL2)* expression using RNA samples from HCT116 cells treated with either *MAPT* or control siRNAs for 48 h (as in Figure 4A). Fold changes were calculated using the ΔΔC_T_ method as described (Devany et al., 2013). Tau knockdown/control ratios were calculated and plotted as shown. Ubiquitin C (UBC) levels were used as endogenous control. The data shown are mean ± SEM from three independent biological experiments. The *P*-values are indicated as *(≤0.05) or **(≤0.005). **(B)** Effect of tau overexpression and phosphorylation at pathological sites on endogenous gene expression of selected mRNA targets analyzed in **(A)**. RNA samples from HCT116 cells transfected with pAc-GFP expression vectors containing WT-Tau or PH-Tau (as in Figure 4B; Alonso et al., 2010) were analyzed as in **(A)**. **(C)** Effect of PARN knockdown on levels of *TP53* and the mRNA targets analyzed in **(A)**. RNA samples were collected from HCT116 cells treated with either PARN or control siRNAs for 48 h. PARN knockdown/control ratios were calculated and plotted as shown. RNA samples were analyzed as in **(A)**. **(D)** siRNA-mediated knockdown of tau decreases *TP53* mRNA levels in HCT116 cells. qRT-PCR analysis of *TP53* using nuclear RNA samples from **(A)**. **(E,F)** WT-Tau overexpression increases *TP53* mRNA levels in non-damaging conditions in HCT116 cells. qRT-PCR analysis of *TP53* using nuclear mRNA samples from **(B)** HCT116 cells transfected with pAc-GFP expression vectors containing WT-Tau and PH-Tau (as in Figure 4B; Alonso et al., 2010); and from **(C)** HCT116 cells transfected with pAc-GFP vectors containing WT-Tau and/or treated with UV (40 Jm^−2^) and allowed to recover for 2 h. Samples were analyzed as in **(A)**.

To further confirm that tau is also part of the deadenylation activator complex we analyzed the effect of tau depletion on the levels of the common mRNA targets of Pin1 and PARN. First, we treated HCT116 cells with *MAPT* siRNA to deplete tau expression (Supplementary Figure S5B), and then checked the levels of common mRNA targets of Pin1 and PARN in these samples by qRT-PCR. We focused our analysis on five of the eight overlapping genes because their function is deregulated in cancer and/or AD, as in the case of *ANXA1* (Lee and Song, 2015; Belvedere et al., 2016). Interestingly, the mRNA levels of four of the five selected genes (*ANXA1, FOS, NOTCH*1 and *PLAGL2*) increased after siRNA-mediated knockdown of tau (Figure 7A). As previously described, mRNA levels of the five selected genes increased after PARN depletion (Figure 7C). We included p53 mRNA levels in our analysis as it is one of the previously described PARN mRNA targets (Devany et al., 2013). Consistent with the data shown in Figure 4, overexpression of WT-Tau in HCT116 cells using the pAcGFP constructs (Supplementary Figure S5C) downregulated the mRNA levels of all five selected genes (Figure 7B), and this effect was lost when PH-Tau was overexpressed. Importantly, as shown in Supplementary Figure S5E, siRNA-mediated knockdown of tau or PARN elongated the poly(A) tail length of *ANXA1* mRNA. These results indicate that tau can induce deadenylation activity of genes whose expression is deregulated in cancer and/or AD, and this activation is not observed in samples from cells expressing tau phosphorylated at residues implicated in neurological disorders. Together these results suggest that tau, Pin1 and PARN have an overlapping role in decreasing the levels of this group of mRNAs, keeping their expression levels low under non-stress conditions.

Our previous study indicated that the p53 signaling pathway is the most significantly affected pathway by PARN knockdown in non-stress conditions (Devany et al., 2013). Importantly, PARN depletion increased not only p53 mRNA but also p53 protein levels, reaching expression levels similar to those observed under DDR. These data are consistent with a feedback loop between p53 and PARN deadenylase, in which PARN deadenylase keeps p53 levels low in non-stress conditions by destabilizing p53 mRNA, and the UV-induced increase in p53 activates PARN, regulating gene expression during DDR (Devany et al., 2013). Our data indicate that siRNA-mediated knockdown of tau decreases p53 protein expression levels before and after UV irradiation in HCT116 cells (Figure 4A), while WT-Tau overexpression increased p53 levels in those conditions (Figure 4B). To further analyze this observation, we determined that tau depletion decreased the levels of *TP53* mRNA under non-stress stress conditions (Figure 7D). *TP53* mRNA levels were upregulated only when WT-Tau was overexpressed (Figure 7E, Supplementary Figure S5C), and this was lost when the pseudo-phosphorylated tau mutant derivative (PH-Tau) was overexpressed. Furthermore, WT-Tau overexpression increases *TP53* mRNA levels only in non-stress conditions (Figure 7F, Supplementary Figure S5D). Interestingly, samples from HCT116 p53^−/−^ cells showed not only a decreased in tau protein (Figure 1D, Supplementary Figure S1A) but also in *MAPT* mRNA levels compared to HCT116 cells independently of stress conditions (Supplementary Figure S6A). Similar results were obtained in samples from p53 depleted HCT116 cells by siRNA treatment (Supplementary Figure S6B). These results suggest an interesting scenario where mRNA and protein levels of p53 and tau might be mutually regulated. Our previous study (Devany et al., 2013) showed that after UV treatment, the changes in the levels of *TP53* mRNA and p53 protein are PARN-independent, indicating there is (are) other mechanism(s) involved in the regulation of p53 expression during DDR. While further studies will be necessary to identify other factors and pathways involved in this mutual regulation, our data is consistent with other studies that point to a functional connection between tau and some p53 isoforms (Wetzel et al., 2008; Pehar et al., 2014).

## Discussion

Previous studies have shown that tau localizes to the nucleus (Brady et al., 1995; Greenwood and Johnson, 1995; Cross et al., 2000; Frost et al., 2014; Bukar Maina et al., 2016) where it binds and protects DNA from oxidative and mild heat stresses, suggesting that tau is involved in DDR (Padmaraju et al., 2010; Sultan et al., 2011). It has been suggested that tau phosphorylation plays a role in its translocation into the nucleus (Alonso et al., 2010; Sarkar et al., 2016) and in DNA binding (Sultan et al., 2011), but tau’s functional roles in the nucleus have not been fully elucidated. The data presented in this study provides evidence of the functional overlapping of nuclear tau with factors involved in mRNA 3′ processing, such as PARN deadenylase and tumor suppressor p53. First, we showed that the distribution of tau forms in the nucleus changed in cells expressing different levels of p53 and under stress conditions (Figure 1). We then determined that tau forms (a) complex(es) with both PARN and p53 in the nucleus under non-stress and DNA damaging conditions (Figures 2, 3). Furthermore, we showed that tau activates PARN-mediated nuclear deadenylation (Figures 4, 5); this activation is stronger in the presence of p53 and reduced by tau phosphorylation at pathological sites (Alonso et al., 2010). Interestingly, we identified Pin1, whose substrates are both tau and p53 in their phosphorylated state and is involved in their isomerization (Lu et al., 1999; Zacchi et al., 2002; Zheng et al., 2002; Kimura et al., 2013), as an additional factor of the deadenylation activation complex together with tau, p53, and PARN (Figures 2, 6). Lastly, we determined that a group of genes with reported connections to cancer and/or AD are regulated by PARN, Pin1 and tau (Figure 7), further supporting the idea of the functional interactions of these factors. Altogether, these studies reveal novel biological roles of tau in mRNA 3′ end processing, specifically deadenylation, in the nucleus; and these functions are modified by tau phosphorylation. Thus, our results suggest that this functional interaction between factors involved in mRNA 3′ end processing, neuropathies and cancer may affect the cellular transcriptome.

In addition, these results are consistent with previous studies that show that tau can also participate in different aspects of RNA metabolism through direct association with RBPs. For example, tau interacts with ribosomes and regulates translation (Meier et al., 2016), and also contributes to the formation of SGs (Vanderweyde et al., 2012, 2016). These cytoplasmic functional interactions are affected in AD (Meier et al., 2016; Vanderweyde et al., 2016; Apicco et al., 2018). In fact, throughout the progression of AD, the colocalization of tau with SGs enriched in T-cell intracellular antigen-1 (TIA-1) and tristetraprolin (TTP) changes. Interestingly, both TIA-1 and TTP can bind PH-Tau (Vanderweyde et al., 2012), and TTP stimulates PARN-mediated deadenylation of AU-rich (ARE)-containing mRNA substrates (Lai et al., 2003) in SG (Zurla et al., 2011). As direct TTP-PARN interactions have not been elucidated, this interaction might require bridging factors. Future studies will be important to determine whether tau might be involved in the TTP-PARN interaction resulting in the regulation of PARN activity and to test whether the appearance of pathological forms of tau and the progression of AD affects this regulation.

Tau phosphorylation has been suggested to be an important modifier of its function and the induction of toxicity in neurons. Different studies have shown that tau can be both phosphorylated and dephosphorylated in the nucleus, most likely depending on cell type and localization in the nucleus, with most of non-phosphorylated tau located in the nucleolus (reviewed in Bukar Maina et al., 2016). Tau forms found in the nucleus of healthy cells are mainly non-phosphorylated and we showed that nuclear tau can associate in (a) complex(es) with PARN/p53/Pin1 during DDR (Figure 2), activate PARN-mediated deadenylation (Figures 4, 5), and protect the cells by inducing expression of genes involved in this response (Figure 7). As AD progresses, studies have shown that while low levels of PH-Tau expression do not induce large aggregates, they allow PH-Tau translocation into the nucleus (Alonso et al., 2010; Sarkar et al., 2016). In the nucleus, tau phosphorylation affects its ability to bind and protect DNA during heat stress (Sultan et al., 2011; Qi et al., 2015) and, as shown here, to regulate PARN-mediated deadenylation and steady-state levels of target mRNAs. When the levels of PH-Tau increase, aggregates and SGs enriched in PH-Tau are formed in the cytoplasm preventing tau translocation into the nucleus (Vanderweyde et al., 2012; Di et al., 2016). As TTP can bind both PARN and PH-Tau in SGs, it is possible that PARN might get trapped and inhibited in SGs affecting the transcriptome during the progression of AD.

Based on the results presented here and in our previous studies (Cevher et al., 2010; Devany et al., 2013), we propose a model for the regulation of mRNA steady-state levels by the functional overlapping of PARN, Pin1, p53 and tau during the progression of tauopathies. In non-pathological conditions, p53 and PH-Tau expression is low. Nuclear WT-Tau forms are in complex(es) with Pin1 and PARN, resulting in the activation of PARN-mediated deadenylation and downregulation of target mRNAs such as *FOS*, *ANXA1*, *NOTCH1* and *PLAGL2*. Interestingly, these targets are ARE-containing mRNA transcripts, supporting previous studies showing that PARN can promote deadenylation of ARE-containing mRNAs (Moraes et al., 2006) and keep the levels of those mRNAs low under non-pathological conditions (Cevher et al., 2010). In addition, it has been reported that Pin1 also regulates mRNA steady-state levels of ARE-containing transcripts by interacting with specific RBPs and regulating complex dissociation (Krishnan et al., 2014). Under pathological conditions, the levels of PH-Tau in the nucleus increase. As PH-Tau that is translocated to the nucleus cannot fully active PARN deadenylase (Figures 4, 5) and most of PARN/Pin1/p53 complex is formed without tau (Figure 3A), nuclear deadenylation is not fully active (Figure 4B). The general outcome in this scenario is the deregulation of the transcriptome, and hence gene expression, in pathological conditions. Vanderweyde et al. (2012) showed that under disease/pathological conditions, as in AD, the levels of abnormal tau are high and localized in cytoplasmic SGs. Our co-localization assays showed that PARN distribution changed in mice expressing PH-Tau (Figures 3C,c,d), showing accumulation of PARN in the perinuclear area and co-localization with PH-Tau staining (Figure 3C). We propose a model where abnormal tau, such as PH-Tau, captures PARN deadenylase in SGs resulting in the reduced levels of nuclear deadenylation.

The findings presented here support the previously described idea of an inverse relationship between cancer and AD (Driver, 2014; Nudelman et al., 2014). Cancer survivors have a lower risk of developing AD than individuals of the same age without a cancer history; and likewise, patients with AD have a lower risk of developing cancer (Driver, 2014). Reports studying this have suggested that these two diseases may share common biological pathways deregulated in opposite directions (Shafi, 2016). Consistent with this, the target mRNAs analyzed here were selected as they have been previously reported being deregulated in cancer and/or AD models. For example, Annexin A1 (encoded by *ANXA1*) is a Ca(2+)-regulated phospholipid-binding protein that is overexpressed in many cancers such as breast, pancreatic, and melanoma (Boudhraa et al., 2014; Belvedere et al., 2016; Shin et al., 2016), having many roles in the progression of this disease by localizing to different cell compartments (Boudhraa et al., 2016). Interestingly, Annexin A1 also has roles in anti-inflammatory and apoptosis responses, its regulation is important for UV-induced damage response, and it is fundamental for brain homeostasis (McArthur et al., 2010; Park et al., 2015). Furthermore, in a genome-wide pathway analysis, *ANXA1* was identified as a potential gene involved in deregulated pathways that may contribute to AD susceptibility (Lee and Song, 2015). The other three genes affected by tau expression have also been linked to these diseases. *FOS* is a proto-oncogene encoding a transcription factor overexpressed in a variety of cancers, including thyroid, bladder and pancreatic (Kataki et al., 2003; Li et al., 2013; Guo et al., 2015). Interestingly, Fos is significantly decreased in AD mice models and is involved in memory formation (Rodriguez-Ortiz et al., 2014). Notch1, a single trans-membrane receptor crucial for T cell development (Takahashi et al., 2007), is deregulated in mammary tumorigenesis. Notch1 induces Pin1 transcription (Rustighi et al., 2009). Interestingly, Pin1/p53-null mice have higher amounts of Notch1 than p53-null or wild-type mice (Takahashi et al., 2007), suggesting that Pin1 downregulates Notch1 expression. Additionally, Notch1 expression is altered in sporadic AD (Berezovska et al., 1998). Finally, proto-oncogene pleomorphic adenoma gene-like 2 (*PLAGL2*) has been implicated in a variety of cancers, including leukemia, gastrointestinal, colon, lung, and prostate (Yang et al., 2011; Hanks and Gauss, 2012; Liu et al., 2014; Guo et al., 2016). *PLAG1*, a *PLAG* gene family member that is very closely related to *PLAGL2* both structurally and functionally (Van Dyck et al., 2007) is a transcription factor that has been associated to single-nucleotide polymorphisms (SNPs) identified in a genome-wide association study using AD databases (Liu et al., 2018).

Furthermore, the list of potential common factors includes Pin1, as a factor involved in cell cycle control and protein folding (Driver et al., 2015), and p53, as a major regulator of apoptosis (Shafi, 2016). Indeed, Pin1 plays opposite roles in the pathogenesis of both diseases, being overexpressed in many cancers and inhibited in AD (Driver et al., 2015). On the other hand, p53 also prevents neurodegeneration in Drosophila models of tauopathies by regulating synaptic gene expression (Merlo et al., 2014). In fact, p53 and apoptosis pathways are upregulated in AD, and tau phosphorylation is indirectly stimulated by p53 (Shafi, 2016). Our results indicate that Pin1, p53 and tau play a role in regulating gene expression by functionally interacting with mRNA 3′ end processing factors, such as PARN, affecting the transcriptome in different cellular conditions. In addition, transcriptomic analyses performed in samples from central nervous system (CNS) disorders and cancers showed expression deregulation in opposite directions at the level of pathways (e.g., genes downregulated in CNS disorders are upregulated in cancers), further supporting the idea of an inverse incidence of both disorders (Ibáñez et al., 2014). Interestingly, Pin1 expression and the p53 pathway show deregulations in opposite directions. While further studies are necessary, it is possible that these changes in the transcriptome might be due to changes in the regulation of mRNA 3′ processing during the onset and development of these diseases.

Our study provides evidence of a functional link between PARN deadenylase, tau, p53 and Pin1, potentially resulting in changes in the expression of transcripts deregulated in AD and/or cancer and in the cellular transcriptome. The data presented here contribute to a better understanding of the association between cancer and AD, offering new opportunities for treatment and/or prevention of both diseases.

## Data Availability Statement

The raw data supporting the conclusions of this manuscript will be made available by the authors, without undue reservation, to any qualified researcher.

## Ethics Statement

The animal study was reviewed and approved by College of Staten Island (CSI) Human & Animal Research Protection Program Office (HARPPO) and written informed consent was obtained from the owners for the participation of their animals in this study.

## Author Contributions

JB, SV and MO performed most of the experiments, with the help of PK, MM and GA. VM and AM prepared WT-Tau and PH-Tau constructs and performed immunofluorescence. DL performed immunofluorescence. JB, AA and FK wrote the manuscript, assisted by SV. AA and FK conceived and financed the study with the help of JB. We all read and approved the final manuscript.

## Conflict of Interest

The authors declare that the research was conducted in the absence of any commercial or financial relationships that could be construed as a potential conflict of interest.
