# Supplementary material for: Nuclear Tau, p53 and Pin1 Regulate PARN-Mediated Deadenylation and Gene Expression
Source: Front Mol Neurosci. 2019 Oct 15;12:242. doi: 10.3389/fnmol.2019.00242 (PMC6843027; doi:10.3389/fnmol.2019.00242)
Supplement: Supplementary file 1 [file Presentation_1.pdf]

Baquero et al., Supplementary 1

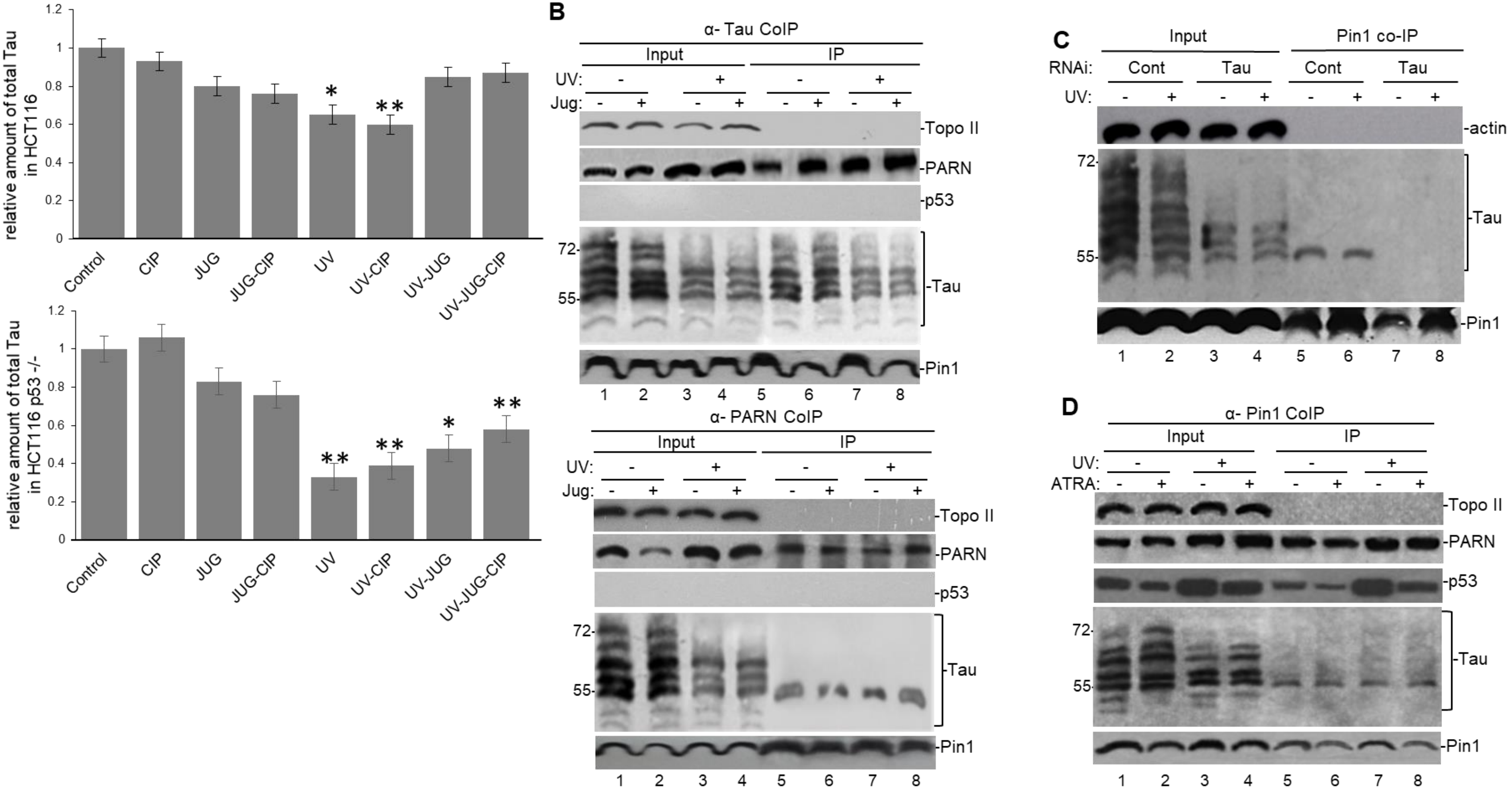

Figure S1. A) Total amount of tau in cellular conditions analyzed in Figures 1B (HCT116 cells) and 1D (HCT116 p53 -/- cells). Addition of band intensities of all tau forms in each condition were plotted. Total tau expression in control conditions (no UV/no juglone/no CIP) were arbitrarily set at 1.0. The quantification of three independent biological assays is shown. Quantifications were done using ImageJ software (http://rsb.info.nih.gov/ij/). The *P* values are indicated as * (≤0.05) or ** (≤0.005). **B)** Tau, Pin1, and PARN form (a) complex(es) independent of p53 expression. Endogenous-immunoprecipitation (e-IP) assays with antibodies against tau (top) or PARN (bottom) were performed using NEs of HCT116 p53 -/- cells treated with 5 μM Juglone for 2 h, and/or exposed to UV irradiation (40 Jm^{-2}) and allowed to recover for 2 h. NEs were treated with RNase A. Equivalent amounts of the pellets (IP) were analyzed by SDS-PAGE, and proteins were detected by Western blot analysis using the indicated antibodies. 10% of the NEs used in the e-IP assays are shown as input. Representative e-IP reactions from three independent biological assays are shown. **C)** Pin1-tau interaction is not detected in *MAPT* siRNA treated cells. Endogenous-immunoprecipitation (e-IP) assays with Pin1 polyclonal antibody were performed using NEs of HCT116 cells treated with either *MAPT* or control siRNAs for 48 h, and/or exposed to UV irradiation (40 Jm^{-2}) and allowed to recover for 2 h. Samples were analyzed as in (B). **D)** Tau can form (a) complex(es) with p53 and PARN in NEs of HCT116 cells treated with Pin1-inhibitor ATRA independently of DNA damaging conditions. e-IP assays with Pin1 polyclonal antibody were performed using NEs of HCT116 cells treated with ATRA (1 μM) for 72 h, and/or exposed to UV irradiation (40 Jm^{-2}) and allowed to recover for 2 h. NEs were treated with RNase A. Samples were analyzed as in (B).

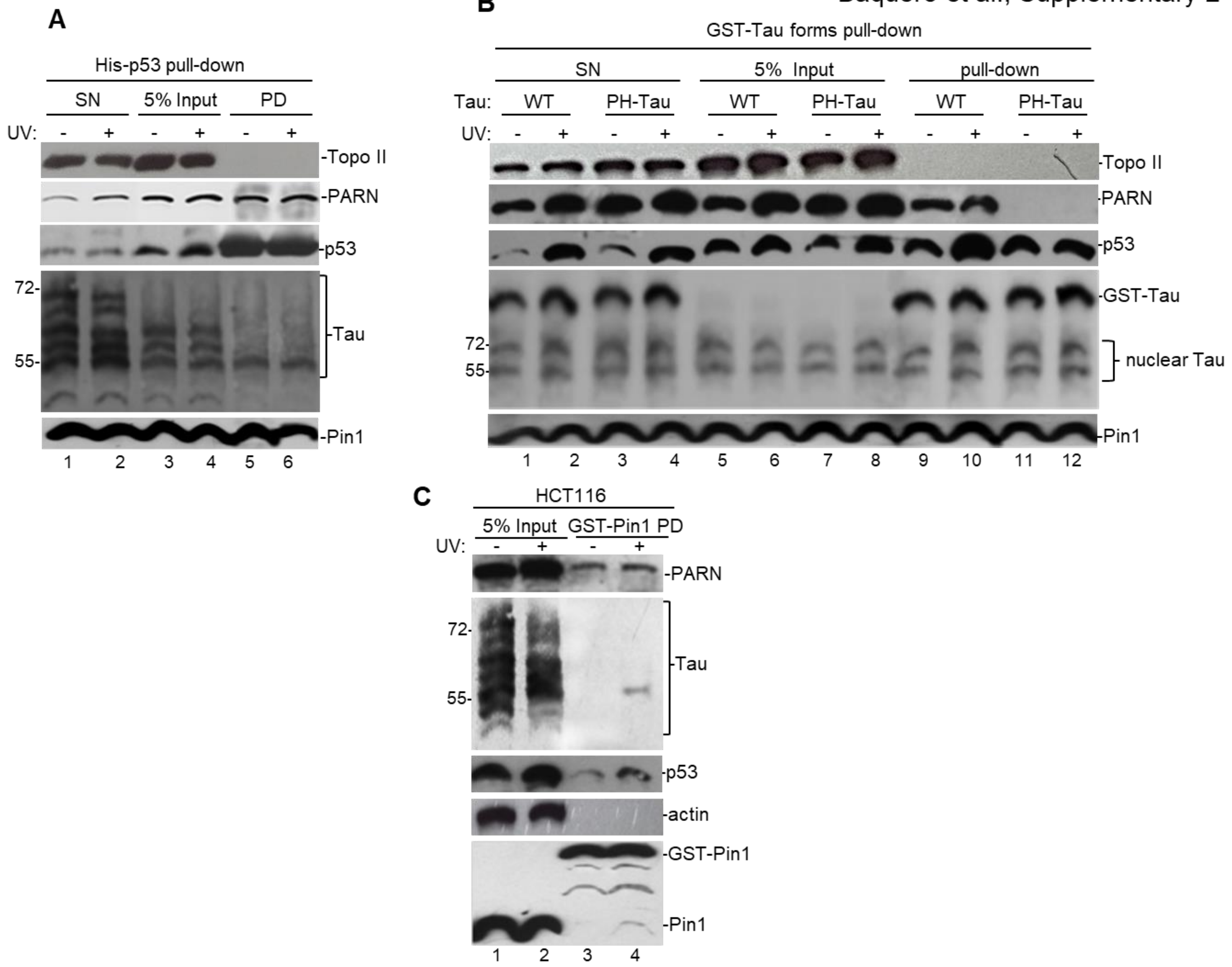
A
His-p53 pull-down
SN
5% Input
PD
UV: - + - + - +
-Topo II
-PARN
-p53
72-
55-
-Tau
-Pin1
1 2 3 4 5 6
B
GST-Tau forms pull-down
SN
5% Input
pull-down
Tau: WT PH-Tau WT PH-Tau WT PH-Tau
UV: - + - + - + - + - + - +
-Topo II
-PARN
-p53
-GST-Tau
72-
55-
nuclear Tau
-Pin1
1 2 3 4 5 6 7 8 9 10 11 12
C
HCT116
5% Input
GST-Pin1 PD
UV: - + - +
-PARN
72-
55-
-Tau
-p53
-actin
-GST-Pin1
-Pin1
1 2 3 4

Figure S2: p53 expression and tau phosphorylation at pathological sites affect complex(es) formed by PARN, Pin1 and tau. **A)** Immobilized His-p53 on nickel beads and **B)** GST-WT-Tau or GST-PH-Tau on glutathione-Sepharose beads (as in Figure 3A) were incubated with NEs from HCT116 cells. Cells were exposed to UV irradiation (40 Jm^{-2}) and allowed to recover for 2 h. NEs were treated with RNase A. Equivalent amounts of the pellets (PD) were resolved by SDS-PAGE, and proteins were detected by Western blotting using indicated antibodies. 5% of the NEs used in the pull-down reactions are shown as input. Representative pull-down reactions from three independent biological assays are shown. **C)** Recombinant Pin1 pulls-down tau, PARN and p53 from NEs of HCT-116 cells. Immobilized GST-Pin1 protein on glutathione-Sepharose beads was incubated with NEs of HCT116 cells using. Samples were analyzed by Western blot as in (A).

Baquero et al., Supplementary 3

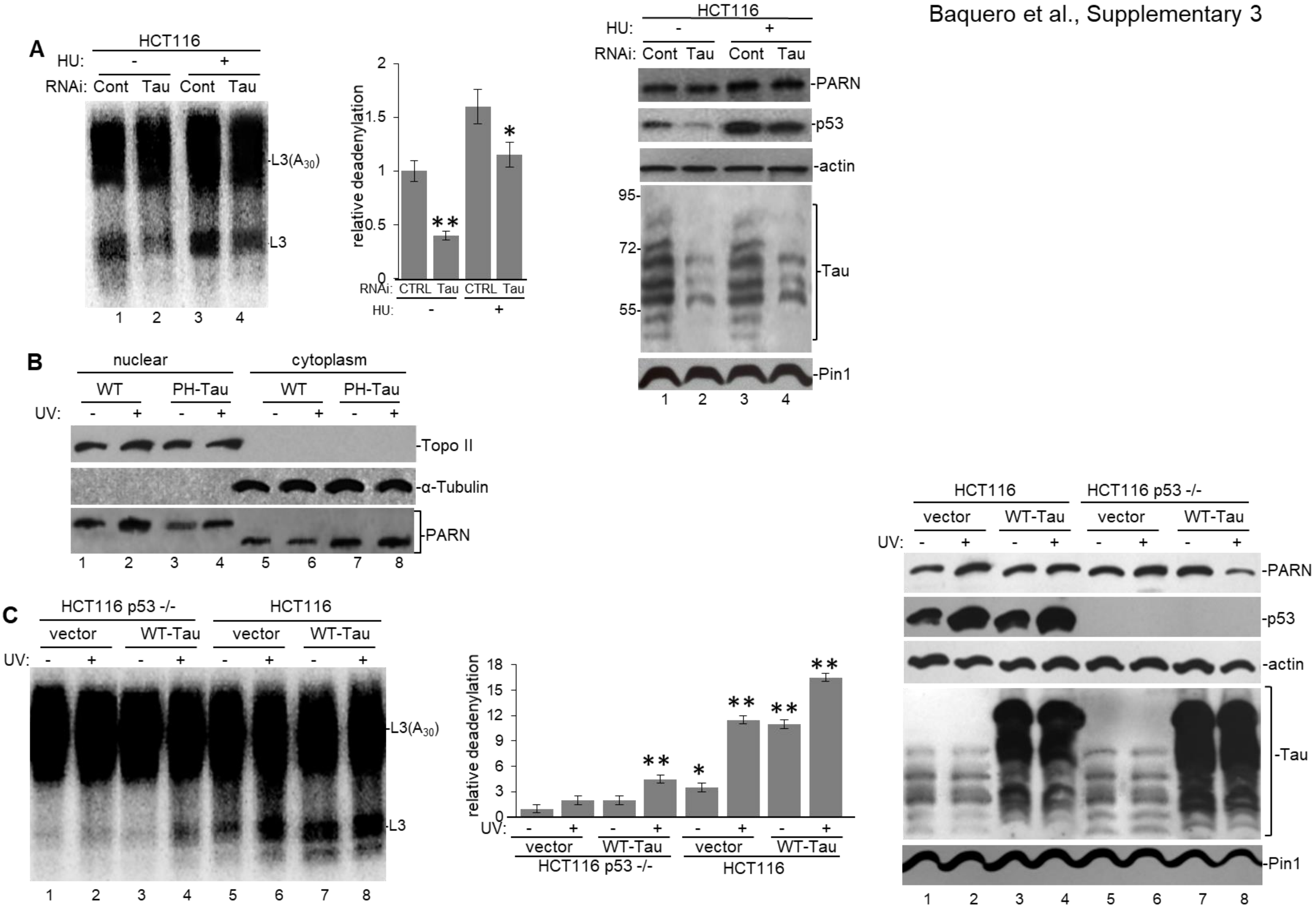

Figure S3. A) Tau depletion inhibits nuclear deadenylation under HU-induced conditions in HCT116 cells. NEs were prepared from HCT116 cells treated with either *MAPT* or control siRNAs for 48 h, and/or treated with HU (1 µM) for 2 h. NEs were used in deadenylation assays as described in Fig. 4 and Cevher et al. (2010). A representative deadenylation reaction from three independent biological assays is shown. Positions of the polyadenylated RNA L3(A_{30}) and the L3 deadenylated product are indicated. Relative deadenylation (RD) levels, calculated as [L3 fragment/(L3 fragment + L3 (A_{30})] ×100, were plotted for each condition. RD for control/no-HU condition was arbitrarily set at 1.0. The means ± standard deviations of RD values are indicated. The *P* values are indicated as * (≤0.05) or ** (≤0.005). Quantifications were done using ImageJ software (http://rsb.info.nih.gov/ij/). Right panel shows expression levels of indicated proteins from NEs tested in deadenylation assays. **B)** Cytosolic PARN levels are enriched in cytoplasmic fractions from cells expressing PH-Tau but not in samples from cells expressing WT-Tau. HCT116 cells were transfected with pAc-GFP expression vectors containing either WT-Tau or PH-Tau (described in Fig. 2), and/or treated with UV (40 Jm^{-2}) and allowed to recover for 2 h. A subcellular fractionation kit was then used to release the protein content of each cellular compartment, as instructed by the manufacturer. Equivalent amounts of total protein content from nuclear and cytoplasmic fractions were analyzed by SDS-PAGE. Antibodies against topoisomerase II (Topo II), and α-Tubulin were used as controls. A polyclonal antibody against PARN deadenylase was used. A representative Western blot from three independent biological assays is shown. **C)** Tau-mediated activation of deadenylation depends on p53 expression. NEs were prepared from HCT116 and HCT116 p53 -/- cells transfected with WT-Tau expressing vector (as in Fig. 4B, Alonso et al. 2010) and/or treated with UV (40 Jm^{-2}) and allowed to recover for 2 h. NEs were used in deadenylation assays as described in (A). RD for control/no-UV condition in HCT116 p53 -/- were arbitrarily set at 1.0.

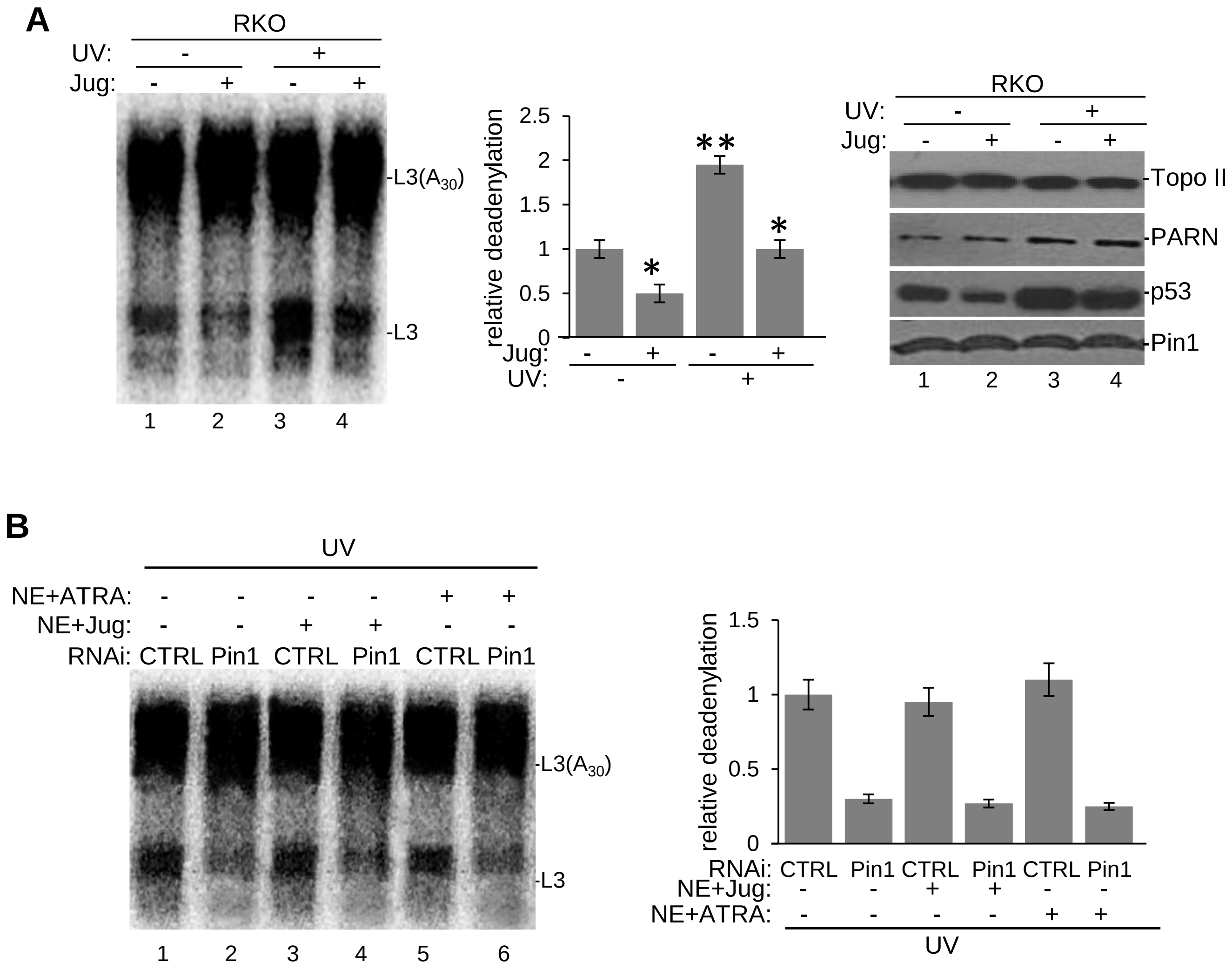
A
RKO
UV: - +
Jug: - + - +
-L3(A30)
-L3
1 2 3 4
relative deadenylation
2.5
2
1.5
1
0.5
0
**
*
*
Jug: - + - +
UV: - +
RKO
UV: - +
Jug: - + - +
-Topo II
-PARN
-p53
-Pin1
1 2 3 4
B
UV
NE+ATRA: - - - - + +
NE+Jug: - - + + - -
RNAi: CTRL Pin1 CTRL Pin1 CTRL Pin1
-L3(A30)
-L3
1 2 3 4 5 6
relative deadenylation
1.5
1
0.5
0
RNAi: CTRL Pin1 CTRL Pin1 CTRL Pin1
NE+Jug: - - + + - -
NE+ATRA: - - - - + +
UV

Figure S4. A) Levels of nuclear deadenylation correlate with Pin1 activity in RKO cells. RKO cells incubated with 5 µM juglone for 2 h and/or treated with UV (40 Jm^{-2}) and allowed to recover for 2 h were used in deadenylation reactions as described in Fig. 4 and Cevher et al. (2010). A representative deadenylation reaction from three independent biological assays is shown. Positions of the polyadenylated RNA L3(A_{30}) and the L3 deadenylated product are indicated. Relative deadenylation (RD) levels, calculated as [L3 fragment/(L3 fragment + L3 (A_{30})] ×100, were plotted for each condition. RD for no-UV/no-juglone condition in RKO cells were arbitrarily set at 1.0. The means ± standard deviations of RD values are indicated. The *P* values are indicated as * (≤0.05) or ** (≤0.005). Quantifications were done using ImageJ software (http://rsb.info.nih.gov/ij/). Right panel shows expression levels of indicated proteins from NEs tested in deadenylation assays. **B)** Addition of Pin1 inhibitors to the deadenylation reaction do not have non-specific ffects, as they only inhibit deadenylation when added to cell cultures. UV-induced activation of nuclear deadenylation is lost in cells with low Pin1 expression, and these deadenylation levels are not affected by addition of Pin1 inhibitors to the reaction. NEs were prepared from HCT116 cells treated with either *MAPT* or control siRNAs for 48 h, and with UV (40 Jm^{-2}) and allowed to recover for 2 h. Either 5 µM juglone or 1 µM ATRA were added to NEs used in deadenylation assays. Reactions were performed at 30°C for 2 h. Samples were analyzed as described in (A). The RD level for samples from control-siRNA/no-inhibitor treated cells was arbitrarily set at 1.0.

A

Genes upregulated in Pin1 siRNA-treated cells (Krishna et al., 2014)	Fold Change in PARN siRNA-treated cells (Devany el al., 2013)	Candidate
*Fos	1.28	Yes
ILG	-1.59	No
NOV	1.1	Yes
IL11	-1.25	No
SLC30A1	-1.11	No
HIC2	-1.13	No
*ANXA1	1.78	Yes
GLS	-1.17	No
*BIRC3	1.18	Yes
EPHA2	-1.34	No
LEPREL1	-1.51	No
C9ORF3	1.19	Yes
PGM2L1	-1.72	No
*NOTCH1	1.38	Yes
RGL1	-1.25	No
*PLAGL2	1.99	Yes
COL12A1	1.07	Yes

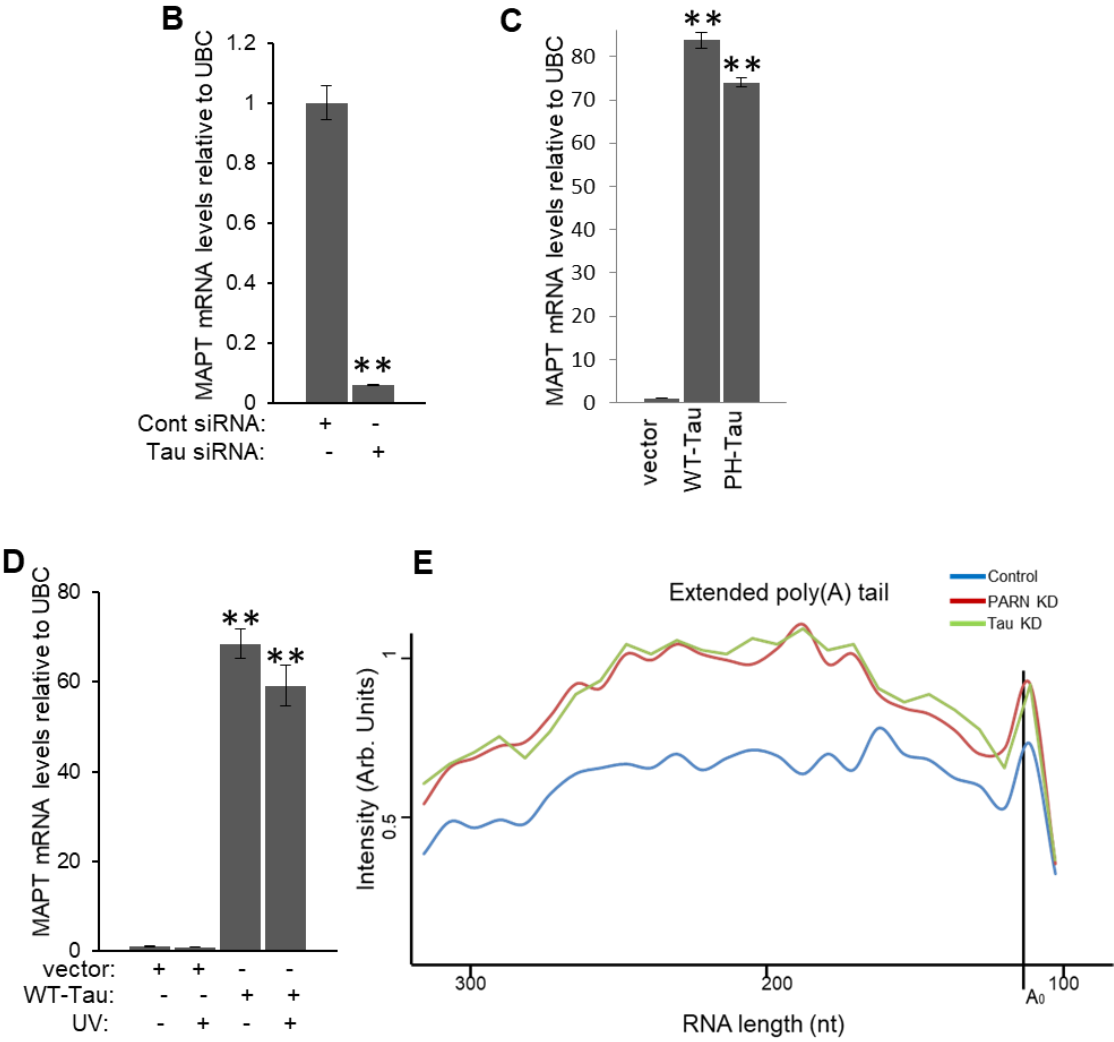

Figure S5: A) Pin1 and PARN common mRNA targets. Genes reported as upregulated after Pin1 siRNA-mediated knockdown (Krishnan et al., 2014) were checked for fold changes after PARN knockdown (Devany et al., 2013). The five genes selected for qRT-PCR analysis in HCT116 cells are indicated with an asterisk. **B-D)** qRT-PCR analysis of *MAPT* mRNA levels using nuclear RNA samples from HCT116 cells treated under different conditions; B) Samples analyzed in Fig. 4A and 7A; C-D) Samples analyzed in Fig. 4B and 7C. Fold changes were calculated using the $\Delta\Delta C_T$ method as in Devany et al. (2013). Ubiquitin C (UBC) was used as endogenous control. The data shown are mean ± s.e.m from three independent biological experiments. The *P* values are indicated as ** (≤0.005). **E)** Quantification of poly(A) tail length of ANXA1 transcript was done by obtaining the density profile of control, PARN KD and tau KD lanes using ImageJ software.

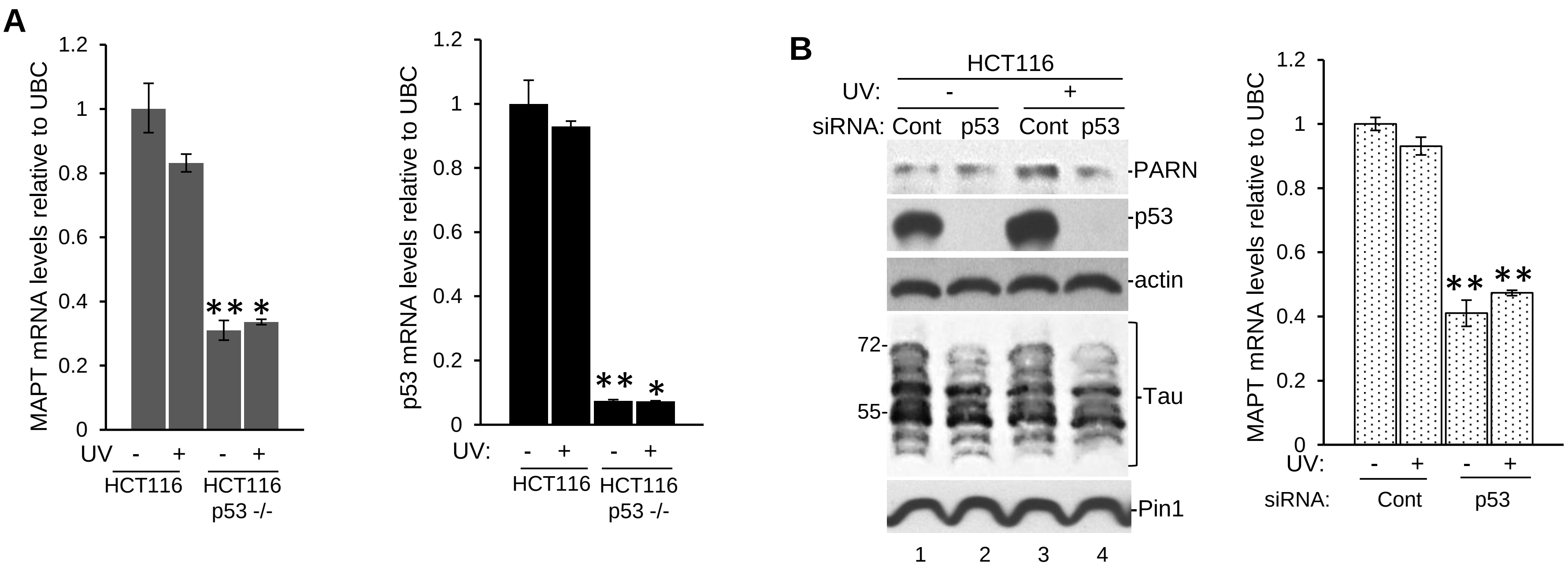
A
MAPT mRNA levels relative to UBC
1.2
1
0.8
0.6
0.4
0.2
0
UV - + - +
HCT116
HCT116
p53 -/-
p53 mRNA levels relative to UBC
UV: - + - +
B
HCT116
UV: - +
siRNA: Cont p53 Cont p53
-PARN
-p53
-actin
72-
55-
-Tau
-Pin1
1 2 3 4
MAPT mRNA levels relative to UBC
UV: - + - +
siRNA: Cont p53

Figure S6: p53 expression increases tau mRNA and protein levels. A) *MAPT* mRNA levels are higher in cells expressing p53. qRT-PCR analysis of *MAPT* (left) and *TP53* (right) mRNAs using nuclear RNA samples from HCT116 and HCT116 p53 -/- cells treated with UV (40 Jm^{-2}) and allowed to recover for 2 h. Fold changes were calculated using the $\Delta\Delta C_T$ method as in Devany et al. (2013). UBC was used as endogenous control. The data shown are mean ± s.e.m from three independent biological experiments. The *P* values are indicated as * (≤0.05) or ** (≤0.005). **B)** p53 knockdown decreases nuclear tau expression and changes nuclear tau patterns in non-stress and UV-induced conditions. NEs from HCT116 cells treated with p53/control siRNAs for 48 h, and/or treated with UV (40 Jm^{-2}) and allowed to recover for 2 h were monitored by Western blotting (left panel). MAPT mRNA levels were also analyzed as in (A). Equivalent amounts of total protein from each sample were analyzed by SDS-PAGE, and tau forms were detected using monoclonal Tau-13 antibody. Actin was used as loading control.
